# Anti-cancer immune effect of human colorectal cancer neoantigen peptide based on MHC class I molecular affinity screening

**DOI:** 10.3389/fimmu.2024.1473145

**Published:** 2024-10-16

**Authors:** Siyu Zhang, Changxin Huang, Yongqiang Li, Zhaoyang Li, Ying Zhu, Lili Yang, Haokun Hu, Quan Sun, Mengmeng Liu, Songqiang Cao

**Affiliations:** ^1^ Department of Oncology, Affiliated Hospital of Hangzhou Normal University, Hangzhou, China; ^2^ Department of Clinical Hematology and Transfusion, The First Affiliated Hospital of Zhejiang Chinese Medical University, Hangzhou, China; ^3^ Department of Psychiatry and Psychology, 155 Hospital of Kaifeng City, Kaifeng, China; ^4^ Department of Urology, Huaihe Hospital of Henan University, Kaifeng, China

**Keywords:** tumor immunotherapy, tumor vaccine, neoantigen, MHC molecular affinity, colorectal cancer

## Abstract

**Background:**

Tumor antigen peptide vaccines have shown remarkable efficacy, safety, and reliability in recent studies. However, the screening process for immunopotent antigenic peptides is cumbersome, limiting their widespread application. Identifying neoantigen peptides that can effectively trigger an immune response is crucial for personalized cancer treatment.

**Methods:**

Whole exome sequencing was performed on patient-derived colon cancer cells to predict 9-amino-acid (9aa) neoantigen peptides. In vitro simulation of endogenous antigen presentation by antigen-presenting cells (dendritic cells) to CD8+ T cells was conducted, aiming to activate the CD8+ immune response to the predicted antigens. The immunological effects of each neoantigen were assessed using flow cytometry and ELISpot assays, while the relationship between neoantigen immunogenicity and MHC molecular affinity was examined.

**Results:**

1. Next-generation sequencing (NGS) predicted 9-amino acid (9aa) neoantigen peptides for subsequent immunological analysis. 2. Higher mDC Levels in Experimental Group: CD11c+CD83+ mature dendritic cells (mDCs) were 96.6% in the experimental group, compared to 0.051% in the control group. CD80 fluorescence intensity was also significantly higher in the experimental group, confirming a greater mDC presence. 3. Neoantigen Peptides Promote CD4+, CD8+ T, and NK Cell Proliferation: After 14 days, flow cytometry showed higher percentages of CD4+ T (37.41% vs 7.8%), CD8+ T (16.67% vs 4.63%), and NK cells (33.09% vs 7.81%) in the experimental group, indicating that the neoantigen peptides induced proliferation of CD4+, CD8+ T cells, and NK cells. 4. The results, analyzed using two-way ANOVA, showed that the standardized T-value for HLA molecular affinity variation in the 1-4 range (Group B) was significantly higher than for ≤1 (Group A, *p* < 0.0001) and >4 (Group C, *p* < 0.05). Regarding HLA-allele genotypes, HLA-Type 1 had a significantly higher standardized T-value than HLA-Type 2 (*p* < 0.05) and HLA-Type 3 (*p* < 0.0001). HLA-Type 1 was identified as the allele associated with the highest T-value.

**Conclusion:**

1. The most immunogenic neoantigens typically exhibit an MHC molecular affinity variation between 1 and 4, indicating that stronger immunogenicity correlates with higher MHC molecular affinity variation. 2. Each patient's HLA molecules were classified into Types 1, 2, and 3, with Type 1 showing the highest binding capacity for neoantigens. Our findings indicate that the most immunogenic neoantigens were associated with HLA Type 1. 3. Neoantigen peptides were shown to activate the proliferation of both CD8+ T-cells and induce proliferation of CD4+ T-cells and NK cells. 4. Variation in MHC molecular affinity and HLA neoantigen genotype are anticipated to serve as valuable variables for screening highly immunogenic neoantigens, facilitating more efficient preparation of effective polypeptide tumor vaccines.

## 1 Introduction

Recent technological developments have enabled significant progress in the prediction and discovery of neoantigens, resulting in numerous studies on tumor vaccines achieving promising outcomes. Currently, there are various types of cancer vaccines used for treatment, including tumor cell vaccines, DC vaccines, T-cell vaccines, DNA vaccines, RNA vaccines, viral vector vaccines, and protein and peptide vaccines (1–4). Each of these approaches has advantages and disadvantages: tumor cell vaccines are significantly limited by patient materials, while DC vaccines, despite being low in toxicity and the only FDA-approved cancer treatment, are not very effective and require additional steps to load and present the antigen. Viral vector vaccines accommodate large gene insertions and readily produce antigens presented by MHC Class I and Class II molecules but carry a high risk of infection or pre-existing immunity. DNA vaccines can deliver a variety of antigens and genes to express both short and long peptides, but it has proven difficult to translate mouse studies into human trials. mRNA vaccines have similar advantages to DNA vaccines, with fewer side effects and less autoimmune issues, but they rapidly degrade and are cleared from the body, reducing their effectiveness. Peptides are easy to produce and cost-effective, allowing multiple peptide sequences to be combined into a single formulation, and the vaccine’s immune effect can be directly monitored by studying the vaccine components. However, whole exon sequencing yields numerous neoantigen mutations, making it challenging to screen for neoantigen polypeptides with high immunogenicity. Therefore, immunogenicity screening becomes the most critical step in the entire process of tumor immunotherapy. Tumor neoantigens have thus become a focal point in this field, offering potential solutions to various challenges encountered in immunotherapy. Studies of individualized tumor vaccines based on tumor neoantigens have also achieved significant success, such as in clinical trials against lymph node metastasis of melanomas (5).

Neoantigens are abnormal proteins arising from genetic mutations in tumor cells. These neoantigens are degraded into short peptides (epitopes) within tumor cells or antigen-presenting cells (APCs). These epitopes bind to major histocompatibility complex (MHC) Class I or Class II molecules on the surface of APCs, forming MHC-polypeptide complexes presented on the APC surface. These complexes are recognized by T-cell receptors (TCRs) on T-cell surfaces, triggering a T-cell immune response and activating effector T-cells to attack and eliminate tumor cells (6). Different tumor types present various quantities and types of neoantigens, but neoantigen types also vary at the individual tumor level; this variation is termed “individualization” (7). Currently, the development of individualized vaccines follows these steps: (1) High-throughput sequencing of the whole exon genome is performed on DNA obtained from tumor tissues. (2) These sequencing results are utilized to predict potential neoantigen peptides and determine the affinity between each peptide and human leukocyte antigen (HLA) Class I molecules. (3) Neoantigen peptides with potential immune effects are screened by their MHC molecular affinity for use in complex immunological experiments, and those with strong immune activity are selected for preparing tumor vaccines for clinical treatment. Individualized tumor vaccines represent a major breakthrough in cancer treatment. Currently, next-generation sequencing (NGS) technologies for neoantigen identification include whole genome sequencing (WGS), whole exome sequencing (WES), and complete transcriptomic sequencing (RNA-seq). Screening for tumor neoantigens is essential for individualized precision therapy. However, since fewer than 10% of tumor cells mutate to form tumor neoantigens, it follows that fewer than 10% of these have sufficient immunogenicity to induce effective specific T-cell immunity, the primary anti-cancer immunity (8).

At present, most experimental studies screen for neoantigen peptides based on their MHC molecular affinity, but not all high-immunogenicity neoantigenic peptides exhibit correspondingly high MHC molecular affinity. Srivastava et al. suggest that neoantigens in transgenic mouse tumor models exhibit very different properties compared to viral antigens, generally displaying very low affinity for MHC Class I antigens (9). An individualized vaccine studied by Patrick A. Ott et al. included only neoantigenic peptides with high MHC molecular affinity variation and achieved significant efficacy when applied to melanoma patients (5). However, screening for neoantigen peptides based on MHC affinity or its variation leads to a high rate of misselection and omission of suitable peptides. Using immunoassays to confirm the immune effect of each antigen peptide in each patient is costly, time-consuming, and labor-intensive, preventing widespread clinical application. Therefore, simple and accurate detection of immunoeffective neoantigen peptides is crucial for the clinical viability of individualized vaccines. In this study, whole exon sequencing was conducted on colon cancer cells obtained from patients. The process of antigen presentation was simulated *in vitro* to analyze and clarify the relationship between the immune activity of neoantigen polypeptides, their HLA Class I molecular affinity, and HLA allele variations. With the rapid commercialization of whole exon sequencing and bioinformatics analysis, new methods for the swift and simple discovery of effective neoantigens have become feasible.

## 2 Materials and methods

### 2.1 Second generation gene sequencing, neoantigen peptide prediction and synthesis

#### 2.1.1 Preparation of specimen

Patients diagnosed with colon cancer by pathology at the affiliated hospital of Hangzhou Normal University and other hospitals between December 2017 and August 2019 were selected and approved by the Medical Ethics Committee of Hangzhou Normal University (Ethics No. 20190052). Patients granted informed consent, and patient information is displayed in **Table 1**. Tumor specimens from six patients with advanced colon cancer confirmed by pathology were obtained after signing an informed consent form. The wax specimens were then dewaxed prior to DNA extraction. DNA was extracted from tissue samples and peripheral blood according to the requirements of each DNA extraction kit (TIANGEN, Beijing, China). The samples were stored at -20° and used within a week.

**Table 1:** The patient's general condition and number.

Number	Gender	Age	Diagnose	Pathological(adenocarcinoma)
1	Male	60	Sigmoid carcinoma	Moderately differentiated
2	Female	47	Transverse colon carcinoma	Highly-Moderately differentiated
3	Male	58	Rectum carcinoma	Moderately differentiated
4	Female	76	Sigmoid carcinoma	Moderately differentiated
5	Female	64	Sigmoid carcinoma	Moderately - Poorly differentiated
6	Female	86	Transverse colon carcinoma	Moderately differentiated

#### 2.1.2 Neoantigen and MHC molecular affinity prediction

The extracted tissue and blood DNA were sent to Shanghai HuiSuan Biotechnology Company, which used a Hiseq2500 sequencing system (Illumina Company) to perform high-throughput sequencing of the human whole exon genome. The gene sequencing data were analyzed using BWA software, with an SNP database of the normal population compiled to evaluate variation. GATK and VarScan software were employed for the identification of gene mutations and variations. Bidirectional sequencing of the whole exome produces hundreds of millions of sequencing fragments, necessitating an average sequencing depth of 300× in the target capture area and a chip sequencing coverage of 99.12% in the target region.

Next-generation sequencing (NGS) and subsequent bioinformatics analysis allow neoantigen peptides of a tumor to be swiftly predicted. Through whole exome sequencing of tumor samples, mutation sites producing abnormal proteins can be screened to obtain the sequences of the mutated proteins. For each set of genome-wide experimental data obtained from patient tumor tissue, neoantigen peptides can be combined with MHC class I molecules and their affinity predicted using software such as NET-MHC 4.0.

### 2.2 Synthesis of neoantigen peptide and non-methylated CpG

The amino acid sequence of each neoantigen peptide was compiled into a table, and 6 mg of each peptide was synthesized by Hangzhou DanGang Biotechnology Co., LTD. Non-methylated CpG adjuvants with a sequence (5’→ 3’) of TCGTCGTTTTGTCGTTTTGTCGTTGGGG were synthesized by Sangon Bioengineering (Shanghai) Co., Ltd.

### 2.3 Preparation of DC vaccine

#### 2.3.1 Cell isolation and culture

Acquiring fresh peripheral blood: After obtaining informed consent from the patient, peripheral blood was extracted using a sampling needle. The amount of blood collected depended on the patient’s physical status and the predicted amount of neoantigen peptide required. The blood was treated with 10 U/mL whole blood heparin to prevent coagulation, then evenly divided into 5 mL tubes and stored at room temperature. The blood was isolated using lymphoid separation solution (Haoyang Co., LTD., Tianjin, China) via the Ficoll density gradient centrifugation method within 24 hours to obtain peripheral blood mononuclear cells (PBMCs). Peripheral blood is added on top of the lymphocyte separation liquid, followed by centrifugation at 450-650g for 20-30 minutes, resulting in the separation of the centrifuge tube contents into four distinct layers from top to bottom. The first layer is the plasma layer, the second layer is a ring-shaped, off-white lymphocyte layer, the third layer is the separation liquid layer, and the fourth layer is the red blood cell layer. The second layer of cyclic grayish white lymphocytes (PBMC) was extracted and cleaned, centrifuged, and then induced and cultured *in vitro*.

Inducing differentiation of DC cells: The PBMCs in RPMI 1640 (Gibco, Grand island, New York, USA) medium with 10% FBS fetal bovine serum (FBS) (Gibco) were adjusted to a density of 2×10^6/mL and added to a 24-well plate for culture. The cells were cultured in an incubator with 5% CO_2_ at 37° for 10 hours, allowed to adhere to the walls, and cleaned by preheating the culture plate twice. Compounds including 100 ng/mL rhGM-CSF (PeProtech, USA), 100 ng/mL rhIL-4 (PeProtech), 50 μg/mL gentamicin (Gibco), 50 μmol/L β-mercaptoethanol (Gibco), and 1% Penicillin-Streptomycin Solution (Gibco) were added to the 10% FBS RPMI 1640 medium. Lymphocyte separation was performed on day 0 (D0), the first day of cell isolation. After allowing the cells to adhere overnight, non-adherent suspended cells were discarded. The adherent cells were washed twice with PBS (Gibco) before fresh medium was added for further culture. Suspended cells were discarded after day 1 (D1). Around day 3 (D3), a half-liquid exchange was performed based on cell growth activity. Around day 5 (D5), the cells began to differentiate into a semi-suspension state, termed immature DCs (imDCs). The specific timing of liquid changes depends on the cell state. If excessive cell growth occurs or if there are many cell fragments due to poor growth, the medium can be replaced ahead of schedule, or the petri dish size can be adjusted appropriately. To determine the differentiation status of the DCs, they were photographed via microscopy on days 1, 2, 3, 5, and 7.

A dose comparison test was necessary before loading the neoantigens. Two neoantigens, ZNF253 and ALPK1, were randomly chosen, and five different doses were prepared: 10 μg, 50 μg, 100 μg, 150 μg, and 200 μg. No neoantigens were added to the control group. According to immunoassays of varying doses of neoantigenic peptide (**Table 2**), the optimal dose is 100 μg. Following the pre-experiment, the change in MHC molecular affinity of each neoantigen peptide sequenced from each patient was categorized into three ranges: <1, 1-4, >4. The HLA allele types of patients were compared, and neoantigens with different intervals and various HLA allele types were randomly selected.

**Table 2:** EISPOT results of the predose loading experiment for neoantigens.

Name of peptides	Load dose of polypeptide/ Chart of ELISPOT /Spot count
**ZNF253**	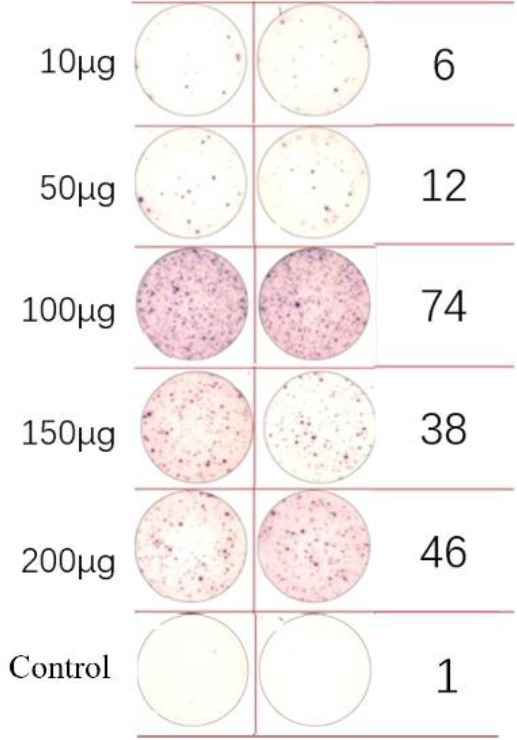
**ALPK1**	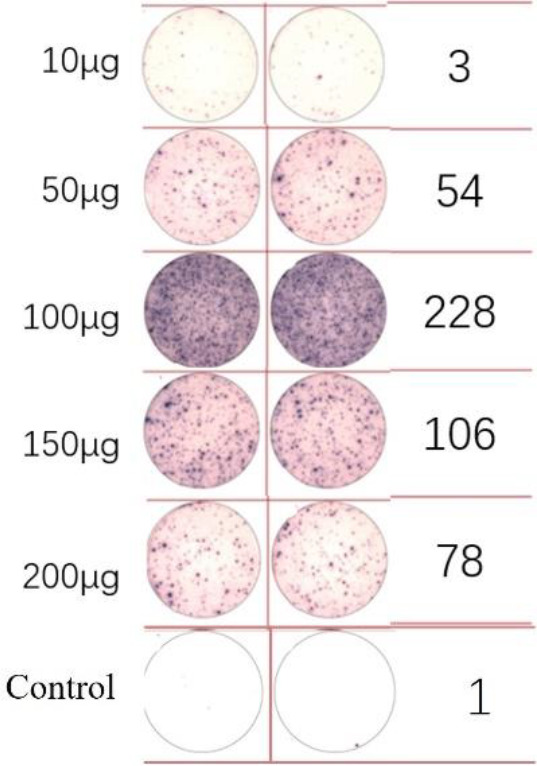

Loading neoantigens: According to the number of imDC cells per well, 100 μg neoantigen peptide, 100 μg CpG adjuvant (Sangon Bioengineering), and 50 ng/mL INF-γ (PeProtech) were added to batches of 1×10^4 DC cells to stimulate differentiation into imDCs. Culture was then continued for 24-48 hours, during which imDCs were loaded with antigen and differentiated into mature DCs (mDCs). The final harvest produced suspended mDCs for use in a DC vaccine.

#### 2.3.2 Identification of mDC via flow cytometry

A sample of a D7 mDC cell suspension was added to a 1.5mL centrifuge tube and centrifuged at 300 G for 5 min. The supernatant was discarded, and the cells were washed three times with PBS, before a 100μL Cell Staining Buffer was added to re-suspend the cells. According to the antibody specification, 5 μl of each of APC anti-human CD83, FITC anti-human Lineage Cocktail (CD3/14/19/20/56), PerCP/Cyanine5.5 anti-human CD80, and PE/Cy7 anti-human CD11c were added to the cell suspension, which was mixed thoroughly and incubated at 4° away from light for 30min before being washed once again with PBS to remove uncombined marker antibodies. 300 μl of Cell Staining Buffer was added for Cell resuspension. BD FACS Diva Software Version 6.13 was employed for computer detection of the resuspended cells.

### 2.4 Stimulation of fresh PBMC by the DC vaccine

To accurately simulate antigen immune response *in vivo*, about 1×10^6^ DC cell suspensions loaded with neoantigen 9aa peptide were taken as stimulation cells in each well – essentially acting as a DC vaccine. Fresh peripheral blood was extracted from the same patient, and PBMCs were once again isolated with lymphoid solution in the previous manner. Suspensions of 5 x 10^6 newly-prepared PBMC cells were added to each of the stimulation cell culture wells, and the culture process continued in a 24-well plate with RPMI 1640 medium (Gibco) containing 10% FBS (Gibco), 60μg/L rhIL-2 (PeProtech), 50μ/ml gentamicin (Gibco), 50μmol/l ß-mercaptoethanol (Sigma) and 1% Penicillin-Streptomycin Solution. Fresh PBMCs were repeatedly stimulated with DC vaccine once every 7 days, for a total of 14 days. In the control group, fresh PBMC was stimulated by DC cultured without neoantigen. All cells were collected for subsequent use of ELISpot enzyme-linked spot detection, and part of the cell suspension was extracted to allow identification of T, B and NK cell percentages via flow cytometry.

### 2.5 Using ELISpot to measure the activity of T cells

Each cell suspension was prepared with two replicate wells. The control group was DC-stimulated PBMC without adding polypeptide culture. At the same time, reagent quality control group was set up, that is, INF-γ (PeProtech) -stimulated cell suspension was used as positive control and pure medium was used as negative control.Each well received 200 μL of RPMI 1640 medium (Gibco) and was incubated at room temperature for about 20 minutes. Subsequently, all mixed cells were collected, centrifuged with RPMI 1640 medium, and resuspended to 200 μL. The test was performed according to Human CD8 +/INF-ELISPOT Kit (R&D Company, USA). The original medium from each well was aspirated, replaced with 200 μL of the mixed cell suspension, and incubated at 37°C in a 5% CO2 incubator for 24 hours. Each well was washed five times with diluted Wash Buffer (R&D) (250-300 μL) and tapped dry. Subsequently, 100 μL of diluted Detection Antibody (R&D) was added to each well and incubated overnight at 2-8°C. The wells were washed again five times with Wash Buffer (R&D) (250-300 μL) and tapped dry. Then, 100 μL of diluted Streptavidin-AP (R&D) was added to each well and incubated at room temperature for 2 hours. After washing twice with PBS (Gibco) (300 μL), the wells were washed again five times with Wash Buffer (R&D) (250-300 μL) and tapped dry. Finally, 100 μL of BCIP/NBT Chromogen (R&D) was added to each well and incubated at room temperature in the dark for 1 hour. Spot analysis was manually conducted using a dedicated automated ELISpot reader for the microplate. ELISPOT Reader (ES-15), Shanghai Fosun Changzheng Medical Science Co., LTD., donated and used by the First Affiliated Hospital of Zhejiang University.

### 2.6 Identifying percentages of T/B/NK cells via flow cytometry

Two tubes of mixed cell suspension, each containing 500 µL volume and a minimum of 10^5 cells per tube, were combined in a 1.5 mL centrifuge tube and centrifuged at 300 G for 5 minutes. The supernatant was discarded before and after washing the cells three times with PBS (Gibco). 10 µL each of CYTO-STAT^®^ tetraCHROME™ CD45-FITC/CD4-RD1/CD8-ECD/CD3-PC5 (Beckman Coulter, Inc., USA) reagent and CYTO-STAT^®^ tetraCHROME™ CD45-FITC/CD56-RD1/CD19-ECD/CD3-PC5 ((Beckman Coulter) reagent were added to each tube. The mixture was thoroughly vortexed and incubated at 20-25°C for 10-12 minutes. A Finally, it was detected by flow cytometer Beckman Navios (Navios, Beckman Inc., USA).

### 2.7 Data analysis and statistics

The T-value data of each patient were standardized by Z-score, converting each patient’s data to the same scale before statistical analysis. Descriptive statistics are presented as mean and standard error of the mean (x̄ ± SE). After testing for homogeneity of variance, two-way ANOVA was used to compare multiple groups across two factors, and an interaction model was created to analyze the interaction between these factors. Differences between variables were considered significant if p < 0.05. The software used for analysis included IBM SPSS Statistics 25 (International Business Machines Corporation, USA), GraphPad Prism version 7.0 (GraphPad Software Inc., USA), and OriginPro 8.0 (OriginLab Corporation, USA).

## 3 Results

### 3.1 Neoantigen prediction

DNA extracted from tumor tissues and the peripheral blood of each patient was sent to Huishang Company in Shanghai for whole-exome sequencing. The neoantigen 9aa peptides predicted by sequencing are displayed in **Table 3**, which contains the names of the mutated genes, the amino acid changes at mutation sites, the amino acid sequence of neoantigen 9-mer peptides, the HLA-I alleles, the MHC molecular affinity of neoantigens (N), the MHC molecular affinity of wild-type peptides (W), and the degree of change in MHC molecular affinity (C), equal to the ratio of W to N.

**Table 3:** Results of whole exon sequencing of tumor specimens.

Patient ID	Gene Name	Mutation	MT Epitope Seq	Peptide Length	HLA Allele	N	W	C
1	ASUN	H/Y	HYKDAHVDF	9	HLA-A*24:02	184.036	3380.08	18.366
1	PFKFB3	E/K	SVTPLASPK	9	HLA-A*03:01	344.702	5681.68	16.483
1	SMC6	E/K	VLYNRSLNK	9	HLA-A*03:01	131.594	2169.05	16.483
1	PPP1R8	E/K	STRAYTLRK	9	HLA-A*03:01	141.948	2339.71	16.483
1	AADAC	G/R	KTIQNLVVR	9	HLA-A*03:01	396.762	4018.93	10.129
1	CDH16	P/L	VSIPELSPL	9	HLA-C*03:04	423.374	3236.91	7.646
1	KANSL1	NI/I	GESSFDINI	9	HLA-B*40:01	200.676	819.146	4.082
1	PTPRF	R/W	QEWIIMYEL	9	HLA-B*40:01	154.782	554.879	3.585
1	ZNF253	T/A	KAFTWPSIL	9	HLA-C*03:04	414.311	932.715	2.251
1	DNAH17	T/M	MMLPTFIAK	9	HLA-A*03:01	123.322	216.465	1.755
1	RNF4	V/M	METAGDEIV	9	HLA-B*40:01	182.056	312.718	1.718
1	ARHGAP15	D/V	KNAIVRLPK	9	HLA-A*03:01	379.957	645.631	1.699
1	SHKBP1	D/N	NLLVSELYR	9	HLA-A*03:01	446.909	711.664	1.592
1	PRICKLE4	D/N	RLETIRNPK	9	HLA-A*03:01	471.752	704.005	1.492
1	BRI3BP	A/V	VYWFLSLTL	9	HLA-A*24:02	265.87	363.864	1.369
1	ARHGAP15	D/Y	LQSYIDFII	9	HLA-B*40:01	271.686	352.242	1.297
1	DLGAP1	R/W	RMWSGSYIK	9	HLA-A*03:01	200.676	254.608	1.269
1	DIEXF	S/F	LIAFPLGLR	9	HLA-A*03:01	427.98	487.316	1.139
1	USP31	G/R	KRFRLPFVL	9	HLA-B*27:05	79.9987	87.2318	1.09
1	TP53	G/S	SSCMSGMNR	9	HLA-A*03:01	437.342	471.752	1.079
1	ZNF493	T/A	EECGKAFSV	9	HLA-B*40:01	367.822	379.957	1.033
1	CYP2A13	A/V	LFKGYGVVF	9	HLA-A*24:02	289.908	299.473	1.033
1	PC	S/T	YFIEVNTRL	9	HLA-A*24:02	487.316	487.316	1
1	ASUN	R/W	HYCTGAYWI	9	HLA-A*24:02	198.516	196.38	0.989
1	RTN4RL2	A/T	RRLLQAPTS	9	HLA-B*27:05	223.607	214.136	0.958
1	KIF13A	V/I	RLRQAITVK	9	HLA-A*03:01	405.442	388.269	0.958
1	SIL1	E/K	VEAIKGGAL	9	HLA-B*40:01	121.995	115.571	0.947
1	ZNF493	E/G	HRIIHTGEK	9	HLA-B*27:05	392.493	337.323	0.859
1	GHRL	M/I	LAIAGSSFL	9	HLA-C*02:02	442.1	359.948	0.814
1	02-Mar	A/T	LRGTQDHLR	9	HLA-B*27:05	476.884	384.09	0.805
1	RCSD1	P/S	ALLPGASSK	9	HLA-A*03:01	482.072	344.702	0.715
1	SLC34A3	R/H	HRVAGSVLK	9	HLA-B*27:05	134.472	95.1188	0.707
1	ZNF362	S/L	RAYLDSASL	9	HLA-C*03:04	306.024	170.612	0.558
1	ATP13A1	R/Q	QEGARVLAL	9	HLA-B*40:01	119.384	44.1204	0.37
2	ZMYM1	NT/X	VIASHSSKF	9	HLA-B*15:01	456.685	10757.6	23.556
2	ARL14EPL	C/F	QQIERQLKF	9	HLA-B*15:01	379.957	5044.15	13.276
2	TARDBP	S/L	SQQNQSGPL	9	HLA-B*15:01	432.636	4106.84	9.493
2	HERC3	YD/Y	EYTFYIPEI	9	HLA-A*24:02	437.342	4151.52	9.493
2	PER2	S/L	CSASGSAAL	9	HLA-B*15:01	432.636	4106.84	9.493
2	CD3G	AV/AF	IVSIFVLAF	9	HLA-B*15:01	442.1	4062.65	9.189
2	OR5H6	G/X	MYLFLEFSL	9	HLA-A*24:02	74.9703	631.809	8.427
2	GLT6D1	P/L	RFAEEYLRL	9	HLA-A*24:02	312.718	2054.82	6.571
2	OR4K5	D/Y	FVYICQASF	9	HLA-B*15:01	271.686	1501.43	5.526
2	SIGLEC8	V/M	MISWIGASM	9	HLA-B*15:01	471.752	2390.89	5.068
2	DOCK4	T/M	CSMSETSGF	9	HLA-B*15:01	302.73	953.119	3.148
2	SNX18	P/A	LQASPQQLY	9	HLA-B*15:01	375.868	1133.26	3.015
2	LMO7	T/M	YMMDDAWKY	9	HLA-B*15:01	432.636	1235.73	2.856
2	SLC25A47	V/L	FYRGLSLPL	9	HLA-A*24:02	437.342	1006.1	2.3
2	MYO9B	V/M	MAAASPSAM	9	HLA-B*15:01	330.102	696.429	2.11
2	CPN2	Q/W	WQGSLGLQY	9	HLA-B*15:01	319.559	585.724	1.833
2	ANKRD12	N/H	HVSNIHSSF	9	HLA-B*15:01	482.072	864.682	1.794
2	TIRAP	R/Q	AYPPELQFM	9	HLA-A*24:02	289.908	461.653	1.592
2	LAMA1	V/L	LEIQANLLF	9	HLA-B*15:01	340.993	482.072	1.414
2	IL10RB	A/V	VFAKGNLTF	9	HLA-A*24:02	271.686	371.823	1.369
2	TNS1	D/N	SYSSPNYSL	9	HLA-A*24:02	271.686	356.074	1.311
2	GLRA3	T/S	YSIRLSLTL	9	HLA-B*15:01	409.852	503.394	1.228
2	CCDC38	D/G	FGSEGSLEF	9	HLA-B*15:01	451.771	548.908	1.215
2	MDH1B	A/V	VYRYESVIW	9	HLA-A*24:02	414.311	497.976	1.202
2	KRT19	A/V	RFGAQLVHI	9	HLA-A*24:02	401.079	482.072	1.202
2	ADH6	C/R	ISRGFSTGF	9	HLA-B*15:01	456.685	537.157	1.176
2	BHLHE22	Q/P	AYLNQGPAI	9	HLA-A*24:02	161.628	188.062	1.164
2	CACHD1	A/V	GYLVVHPTL	9	HLA-A*24:02	277.629	319.559	1.151
2	ULK1	I/V	MYMAPEVVM	9	HLA-A*24:02	192.176	205.066	1.067
2	PLEKHM3	L/S	SYDNTQLQL	9	HLA-C*04:01	492.617	520.002	1.056
2	ITPKA	R/C	ALCGCVPAF	9	HLA-B*15:01	461.653	392.493	0.85
2	TTN	E/K	YQIKSSIDF	9	HLA-B*15:01	115.571	98.257	0.85
2	GLB1L2	A/V	TMVGPALQF	9	HLA-B*15:01	471.752	396.762	0.841
2	ALPK1	T/M	AFSAYMPLF	9	HLA-A*24:02	226.039	182.056	0.805
2	CYP2E1	N/D	PWLQLYDNF	9	HLA-A*24:02	274.641	214.136	0.78
3	MEX3C	AAAAA/A	REAAAAGVL	9	HLA-B*40:06	134.472	5932.98	44.12
3	CLEC4D	R/W	RWLSYFLGL	9	HLA-A*24:02	151.468	2145.7	14.166
3	SLC45A3	ALGPTEPAEGLSA/A	VAEEAAPSL	9	HLA-C*08:01	309.353	2635.42	8.519
3	MYO16	K/M	YEREKAFQM	9	HLA-B*40:06	312.718	1422.36	4.548
3	TKTL2	W/L	GESSEGSVL	9	HLA-B*40:06	456.685	1904.94	4.171
3	METTL21C	L/FX	RFSTDYEFF	9	HLA-A*24:02	216.465	727.232	3.36
3	FLT4	R/L	RFLESTEVI	9	HLA-A*24:02	168.776	514.406	3.048
3	C10orf120	D/Y	QEYLSSASP	9	HLA-B*40:06	482.072	1304.42	2.706
3	CHRDL1	C/Y	RVRCPNVHY	9	HLA-A*30:01	316.12	743.14	2.351
3	OR10K1	N/Y	RYMAICYPL	9	HLA-A*24:02	46.0718	93.0826	2.02
3	CFH	C/F	YQFQNLYQL	9	HLA-B*13:02	352.242	688.934	1.956
3	ALG1L2	L/M	CMPACAVNF	9	HLA-A*24:02	409.852	792.984	1.935
3	CACNA1S	N/D	WFTDFILLF	9	HLA-A*24:02	200.676	263.008	1.311
3	CYP26B1	R/H	KEVMHLFTP	9	HLA-B*40:06	326.549	401.079	1.228
3	APOB	T/N	FERNRQNII	9	HLA-B*13:02	414.311	451.771	1.09
3	COMMD10	A/T	MAVPTALIL	9	HLA-C*08:01	471.752	492.617	1.044
3	PTPRO	E/K	NYFFKFEEF	9	HLA-A*24:02	165.163	168.776	1.022
3	ZNF597	Q/E	SELISHENI	9	HLA-B*13:02	359.948	356.074	0.989
3	PYDC1	A/T	YEDYTAELV	9	HLA-B*40:06	316.12	306.024	0.968
3	CEP97	S/T	NEITFLASL	9	HLA-B*40:06	316.12	306.024	0.968
3	ZBTB39	F/V	IEQTVMYII	9	HLA-B*13:02	323.035	302.73	0.937
3	FMN1	A/T	TSIPPPPPL	9	HLA-C*08:01	388.269	344.702	0.888
3	GATA6	A/V	REPGGYAAV	9	HLA-B*40:06	209.552	184.036	0.878
3	ANKRD26	S/C	AELECGSIA	9	HLA-B*40:06	344.702	296.25	0.859
3	ZNF518B	L/F	KTKQVHFSR	9	HLA-A*30:01	456.685	388.269	0.85
3	MCAM	R/C	CRCSGKQEM	9	HLA-C*06:02	476.884	363.864	0.763
3	RWDD3	IV/MM	KYVKMMEKW	9	HLA-A*24:02	401.079	299.473	0.747
4	PIK3R1	Q/X	YLMWLTQKV	9	HLA-A*02:01	6.71458	216.465	32.238
4	CXCR4	F/L	NLLCKAVHV	9	HLA-A*02:01	15.6149	286.788	18.366
4	MALT1	R/Q	CQATGHPFV	9	HLA-A*02:01	124.664	2032.71	16.306
4	FLT4	T/M	SMLHDAHGN	9	HLA-A*02:01	356.074	5500.22	15.447
4	FAT1	P/L	FPAADELPL	9	HLA-B*15:11	196.38	2936.57	14.953
4	AR	R/W	RLWKCYEAG	9	HLA-A*02:01	451.771	6610.93	14.633
4	RECQL4	R/W	GVWRGTGVL	9	HLA-A*02:01	432.636	6330.92	14.633
4	ATM	Q/X	KMMEVQKSL	9	HLA-A*02:01	53.03	759.397	14.32
4	TBX3	P/L	PLPGLGFAL	9	HLA-A*02:01	178.159	1437.83	8.071
4	BLM	S/I	SIIIGSSSA	9	HLA-A*02:01	446.909	3272.12	7.322
4	PTPRS	A/V	FIHEALLEV	9	HLA-A*02:01	24.0712	105.988	4.403
4	HGF	Q/H	EHHKMRMVL	9	HLA-B*38:01	442.1	1691.19	3.825
4	PTPRT	P/X	QVKFLNPRK	9	HLA-A*30:01	461.653	1709.59	3.703
4	IRS1	R/H	HHCTPGTGL	9	HLA-B*38:01	363.864	1235.73	3.396
4	PTEN	R/C	TIPSQRCYV	9	HLA-A*02:01	312.718	893.209	2.856
4	ACVR1B	R/H	HVHTCIPKV	9	HLA-A*02:01	178.159	497.976	2.795
4	PTPRD	T/M	LLLTFFLRM	9	HLA-A*02:01	66.558	104.847	1.575
4	TSC1	R/H	ALFHHLYGM	9	HLA-A*02:01	17.0267	23.3024	1.369
4	CTNNA1	V/A	PLLALIEAA	9	HLA-A*02:01	148.226	200.676	1.354
4	NKX2-1	A/T	APAPGTASL	9	HLA-B*15:11	492.617	625.01	1.269
4	RB1	I/L	KLERTCELL	9	HLA-A*02:01	221.2	251.868	1.139
4	NF1	R/Q	FMAIQNPLE	9	HLA-A*02:01	344.702	371.823	1.079
4	SDHA	Y/C	SISAQCPVV	9	HLA-A*02:01	130.178	133.025	1.022
4	SOX2	A/P	MHRYDVSPL	9	HLA-B*38:01	446.909	446.909	1
4	TET2	G/V	VTRFHFQQR	9	HLA-A*30:01	437.342	432.636	0.989
4	ANKRD11	A/V	VLASSLIGG	9	HLA-A*02:01	277.629	260.178	0.937
4	SPRED1	H/R	MLYRCMSDS	9	HLA-A*02:01	306.024	268.762	0.878
4	POLD1	D/G	FMVGTDIVG	9	HLA-A*02:01	286.788	230.984	0.805
4	RUNX1	R/H	HILPPCTNA	9	HLA-A*02:01	296.25	226.039	0.763
4	FOXL2	P/S	FLNNSWSLP	9	HLA-A*02:01	192.176	102.603	0.534
5	SLC6A3	G/R	FRPPHYRAY	9	HLA-C*07:02	401.079	1967.79	4.906
5	EVPL	R/I	LLQQLEFAI	9	HLA-A*02:07	48.6329	2664.09	54.78
5	HCN1	P/R	RVDYIFLIV	9	HLA-A*02:07	126.02	560.916	4.451
5	DAPK1	R/H	MVYGGVHIV	9	HLA-A*02:07	23.5559	84.4457	3.585
5	NOC3L	E/A	KLARELREA	9	HLA-A*02:07	151.468	466.676	3.081
5	THAP8	R/H	SCFQWHWGV	9	HLA-A*02:07	196.38	476.884	2.428
5	LOXL4	R/Q	ALGPQGQRL	9	HLA-A*02:07	223.607	379.957	1.699
5	TTN	R/H	KMKPHPIAI	9	HLA-A*02:07	274.641	375.868	1.369
5	FERMT2	K/N	NMKYVKVNV	9	HLA-A*02:07	375.868	487.316	1.297
5	SASH1	S/I	ITLSQVPSL	9	HLA-A*02:07	172.469	218.82	1.269
5	SGMS1	R/Q	AQLQRIMKL	9	HLA-A*02:07	151.468	188.062	1.242
5	ZNF556	R/H	FSCPKSFHA	9	HLA-A*02:07	392.493	471.752	1.202
5	CCDC168	Q/P	HLKPKPKYV	9	HLA-A*02:07	492.618	585.724	1.189
5	TGM3	T/M	KLKPNMPFA	9	HLA-A*02:07	138.909	161.628	1.164
5	POGK	D/N	SMNTNMVII	9	HLA-A*02:07	80.869	94.0952	1.164
5	BPIFB4	D/N	LIDVNTEFL	9	HLA-A*02:07	216.465	251.868	1.164
5	MOS	E/D	RSFWADLNV	9	HLA-A*02:07	414.311	466.676	1.126
5	SETD1A	P/S	DLQDSRCHV	9	HLA-A*02:07	312.718	348.452	1.114
5	BTNL3	S/F	GMIIVFFKF	9	HLA-A*02:07	211.831	223.607	1.056
5	OR2A25	V/A	MLANLLHPA	9	HLA-A*02:07	14.6334	14.9535	1.022
5	OR5B2	M/T	LVFTSSFNI	9	HLA-A*02:07	82.638	81.7487	0.989
5	LAMA5	V/I	LLLIGLALL	9	HLA-A*02:07	18.1687	17.5884	0.968
5	LRRC18	I/M	KVARNCMKI	9	HLA-A*02:07	482.072	451.771	0.937
5	TMEM151A	A/V	VLIRRLQQA	9	HLA-A*02:07	92.0809	86.293	0.937
5	NDUFS7	R/S	SGCDRIVPV	9	HLA-A*02:07	333.693	306.024	0.917
5	DNAH3	A/E	DLAEEMPAL	9	HLA-A*02:07	119.384	103.719	0.869
5	SLC10A4	N/S	TLAPSASSL	9	HLA-A*02:07	119.384	101.499	0.85
5	VSIG10L	M/I	IRSEGDQLL	9	HLA-C*07:02	363.864	296.25	0.814
5	NEUROD1	R/G	GLAKNYIWA	9	HLA-A*02:07	128.777	104.847	0.814
5	TP53	L/R	GLAPPQHRI	9	HLA-A*02:07	100.406	79.1378	0.788
5	CILP2	D/N	YQGNFTIEV	9	HLA-A*02:07	68.7539	52.4594	0.763
6	MMP17	P/PL	PLLPLLLLL	9	HLA-A*02:01	55.9779	1672.99	29.887
6	NEB	S/L	SLVLYKEDV	9	HLA-A*02:01	101.499	2842.78	28.008
6	CACNA1F	R/Q	NQNNNFQTF	9	HLA-B*15:01	188.062	1602.13	8.519
6	ASXL1	QK/Q	KQKKKERTW	9	HLA-B*15:01	5210.57	42971.9	8.247
6	ATP7B	I/F	RINLVLALF	9	HLA-B*15:01	645.631	3307.72	5.123
6	ADAMTS1	V/M	LLAAALLAM	9	HLA-B*15:01	384.09	1946.61	5.068
6	PITPNM2	V/M	KVRAGAVDM	9	HLA-B*15:01	2145.7	10874.7	5.068
6	PIK3CA	H/R	ARHGGWTTK	9	HLA-C*07:02	2390.89	9447.76	3.952
6	TREX2	V/M	AMVRTLQAF	9	HLA-B*15:01	249.158	727.232	2.919
6	IL7R	T/I	LLIISILSF	9	HLA-B*15:01	198.516	573.186	2.887
6	OR1I1	I/F	IFTDSHLHT	9	HLA-C*07:02	7131.08	16405	2.3
6	MMP17	P/PL	LSRLPLLPL	9	HLA-B*15:01	1318.61	2968.51	2.251
6	AMER1	R/H	KGFFSSIRH	9	HLA-B*15:01	9550.53	20813.9	2.179
6	PLEKHA4	P/S	SSSRNTTPY	9	HLA-B*15:01	645.631	1376.93	2.133
6	DLGAP4	R/C	WSTTLLSPC	9	HLA-B*15:01	5681.68	10414	1.833
6	OR51M1	F/L	IHRAIIKLL	9	HLA-C*07:02	2390.89	4242.33	1.774
6	LRRC16B	R/H	RGPSFHRKM	9	HLA-A*02:01	32085.8	50000	1.558
6	LRRC16B	R/H	RGPSFHRKM	9	HLA-B*15:01	5932.98	8478.89	1.429
6	SEMA5A	R/H	CSHDCSRGI	9	HLA-C*07:02	2240.61	3202.08	1.429
6	POM121L12	G/S	SLSPWSLSF	9	HLA-B*15:01	299.473	409.852	1.369
6	RELN	G/S	SSQPVTWAI	9	HLA-B*15:01	3454.02	4526.88	1.311
6	TEX13B	D/E	GLATAGGEW	9	HLA-B*15:01	3848.7	4989.87	1.297
6	RCVRN	A/T	FQSIYTKFF	9	HLA-B*15:01	293.062	371.823	1.269
6	ZNF395	E/V	FLLDVPAPR	9	HLA-B*15:01	6469.41	8119.76	1.255
6	NTRK3	K/T	YRTFTTESD	9	HLA-C*07:02	1485.27	1824.25	1.228
6	ALDH3B2	R/H	TVANHVAWF	9	HLA-B*15:01	819.146	1006.1	1.228
6	LRMP	G/S	MSFLTSQLF	9	HLA-B*15:01	205.066	251.868	1.228
6	POM121L12	G/S	LKPSLSPWS	9	HLA-C*07:02	3529.58	4196.68	1.189
6	RCVRN	A/T	IYTKFFPDT	9	HLA-C*07:02	2579.01	3066.45	1.189
6	MMP21	A/V	ALAEVVRRF	9	HLA-A*02:01	598.537	688.934	1.151
6	LAMA1	Q/H	VGFSCHDCA	9	HLA-A*02:01	3066.45	3454.02	1.126
6	GM2A	S/T	GCIKIAATL	9	HLA-B*40:01	631.81	688.934	1.09
6	IQUB	D/N	CNNLSDLVM	9	HLA-B*15:01	1904.94	2010.83	1.056
6	PITPNM2	V/M	VRAGAVDMV	9	HLA-C*07:02	1584.89	1672.99	1.056
6	SLC26A9	A/T	HTNITSLIF	9	HLA-C*07:02	18279.6	19088.1	1.044
6	RELN	G/S	WYQGFYPAS	9	HLA-C*07:02	2664.09	2722.37	1.022
6	PIK3CA	H/R	RHGGWTTKM	9	HLA-B*15:01	3932.89	3890.57	0.989
6	SLC26A9	A/T	HTNITSLIF	9	HLA-B*15:01	461.653	446.909	0.968
6	GPR83	A/T	DEQSTEAAL	9	HLA-B*40:01	196.38	190.108	0.968
6	GGN	A/V	APAPVAAPI	9	HLA-B*40:01	4018.93	3807.28	0.947
6	MMP21	A/V	ALAEVVRRF	9	HLA-B*15:01	864.682	819.146	0.947
6	SDCCAG3	S/L	LVVQQKRAV	9	HLA-C*07:02	6262.79	5932.98	0.947
6	HNRNPA1	G/E	FGNDGGYEG	9	HLA-A*02:01	5932.98	5560.06	0.937
6	PKHD1L1	N/S	SRSIKIVGE	9	HLA-C*07:02	3848.7	3606.79	0.937
6	NCKAP5	S/C	TLEREVPCS	9	HLA-A*02:01	1946.61	1824.25	0.937
6	GM2A	S/T	GCIKIAATL	9	HLA-B*15:01	2240.61	2099.77	0.937
6	PCDH20	I/V	VISLGSVTL	9	HLA-C*07:02	10302	9447.76	0.917
6	PER3	A/T	TLSTGSPPM	9	HLA-C*07:02	6062.77	5382.48	0.888
6	SPHK2	A/T	HLGTDLVAA	9	HLA-B*15:01	7208.66	6330.92	0.878
6	PER3	A/T	TLSTGSPPM	9	HLA-B*15:01	743.14	652.654	0.878
6	GABRR1	A/T	LQTYFPTTL	9	HLA-B*15:01	1085.26	932.715	0.859
6	NCKAP5	S/C	REVPCSTDG	9	HLA-B*15:01	7446.48	6399.79	0.859
6	GRM4	T/M	KLYIQMTTL	9	HLA-B*40:01	1249.17	1017.05	0.814
6	NEB	S/L	NQKNFSLVL	9	HLA-B*15:01	548.908	442.1	0.805
6	VASH1	V/I	DLRDGGIPF	9	HLA-B*15:01	819.146	631.81	0.771
6	GRM4	T/M	KLYIQMTTL	9	HLA-B*15:01	1209.27	932.715	0.771
6	AIFM3	A/T	RPKEFFRTY	9	HLA-B*15:01	1904.94	1453.47	0.763
6	TTN	A/V	YSLLIAEVY	9	HLA-B*15:01	864.682	652.654	0.755
6	FAT4	V/I	DLNDNAPIF	9	HLA-B*15:01	1517.76	1133.26	0.747
6	TNIP1	M/L	LAMEHPPPL	9	HLA-B*15:01	837.065	560.916	0.67
6	PCDH20	I/V	GSVTLVTGM	9	HLA-B*15:01	1362.11	846.171	0.621
6	OR51M1	F/L	RAIIKLLGL	9	HLA-B*15:01	1884.44	1121.07	0.595
6	PSG1	L/W	LPMTHSWKL	9	HLA-B*15:01	4830.5	2842.78	0.589
6	SETD7	Y/C	ECDTDGRLI	9	HLA-C*07:02	7609.38	1517.76	0.199

### 3.2 The hybrid DC cell vaccine was successfully prepared

**Figure 1:**
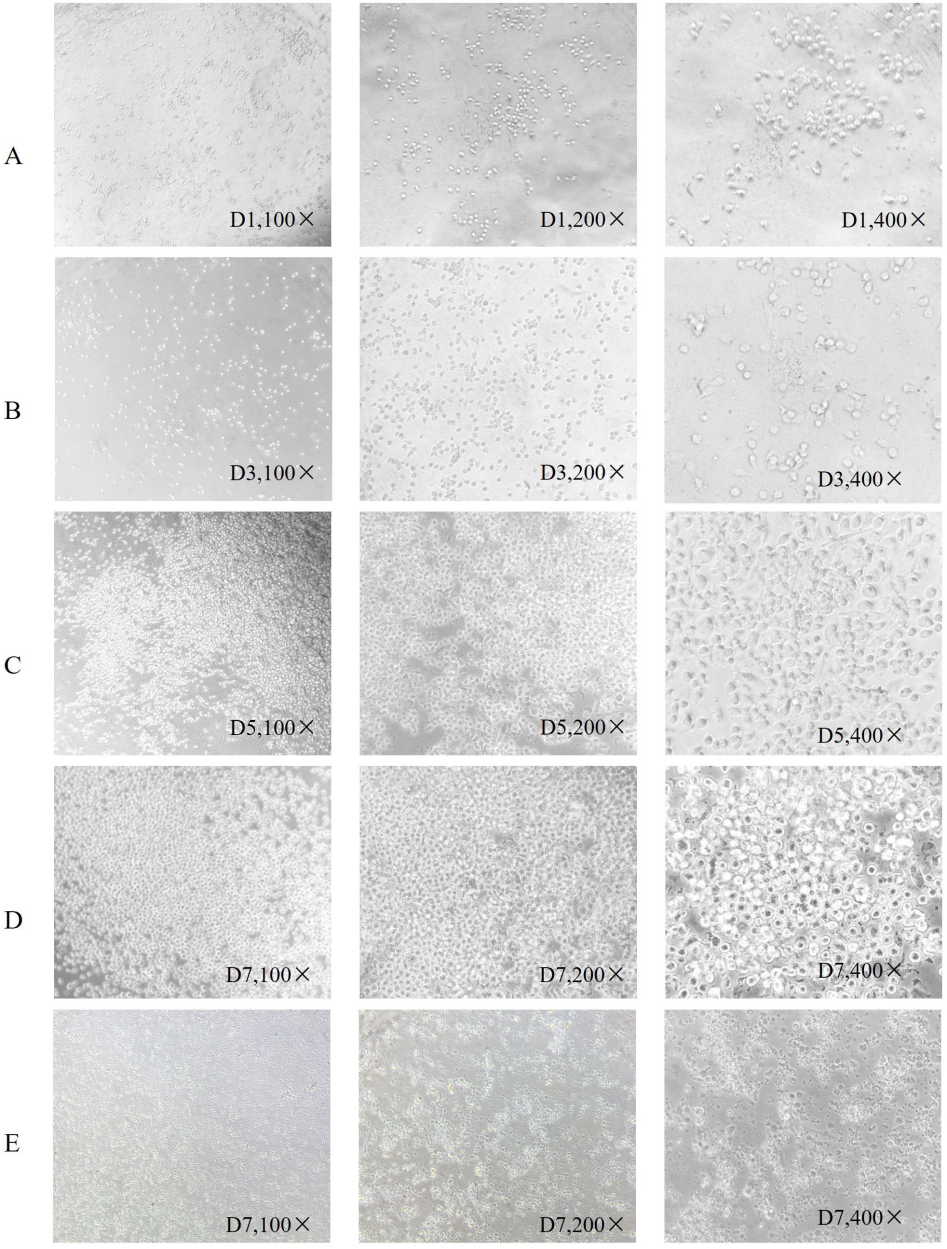
Morphological changes of dendritic cells. Is arranged into groups **(A–E)** with all photographs taken under a light microscope. Group **(A)** shows round monocytes with strong wall attachment after the removal of suspension cells on day D1. Group **(B)** shows adherent cells beginning to grow in clusters and extend pseudopodia on day D3. Group **(C)** shows a variety of cells with different morphology and pseudopodia on day D5. These can be characterized as immature dendritic cells, as the number of cells has significantly increased and some of the cells are suspended. Group **(D)** shows dendritic cells suspended after culture stimulation by CpG adjuvant and neoantigen 9aa peptides. They display short, transparent pseudopodia and are identified as mature dendritic cells. Group **(E)** shows the control group, no neoantigen 9aa peptides was added. The immature dendritic cells gradually die and break into cell fragments.

**Figure 2:**
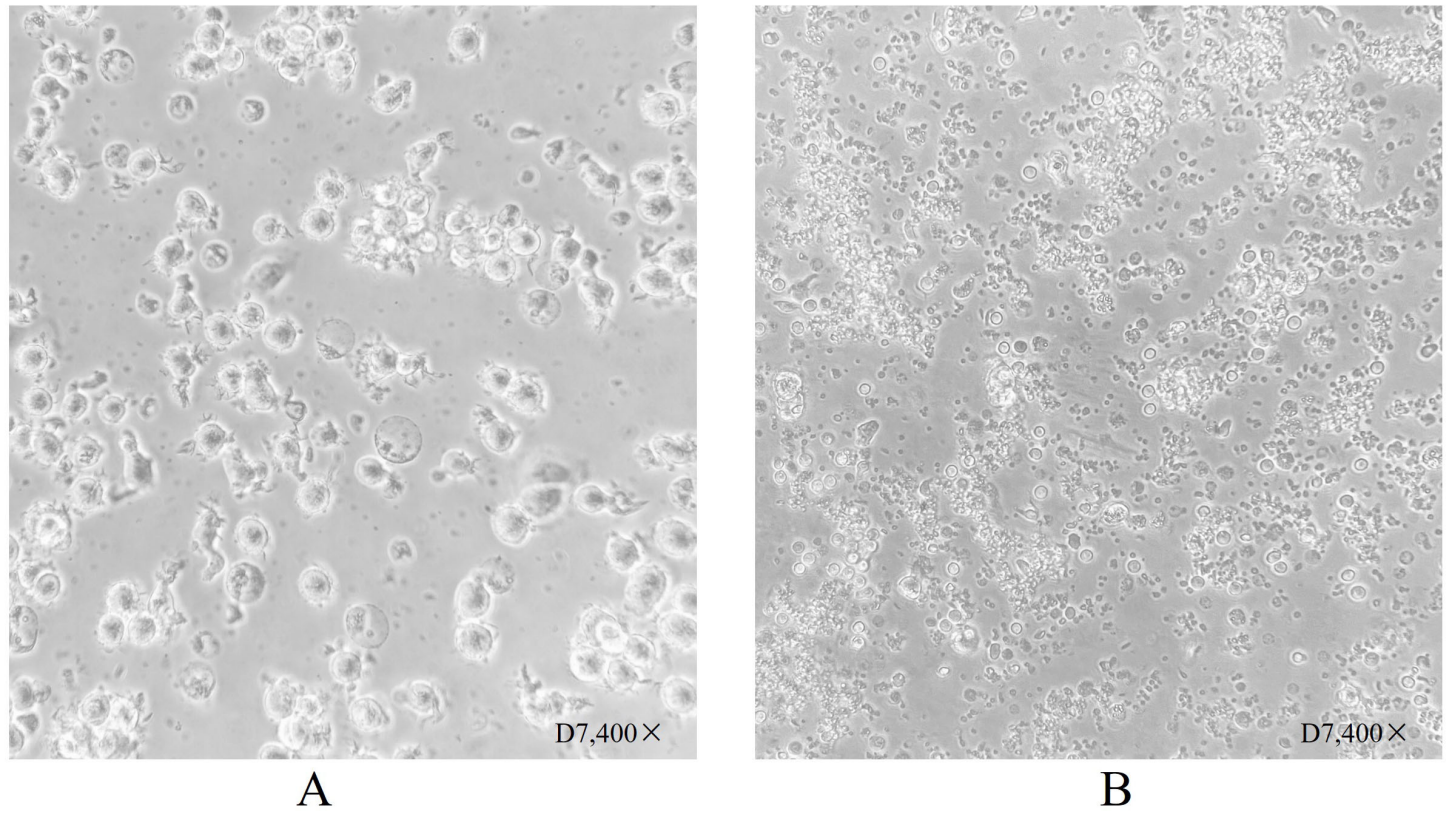
Dendritic cell morphology after 7 days of culture. **(A)** Shows dendritic cells suspended following culture stimulation with CpG adjuvant and neoantigen 9aa peptides on day D7. They display short, transparent pseudopodia, which are identified as mature dendritic cells. **(B)** Shows dendritic cells at day 7 without stimulation by neoantigen 9aa peptides; the immature dendritic cells exhibited gradual apoptosis and lysis into cell fragments.

PBMC differentiation into immature DC was induced by rhGM-CSF and rhIL-4 *in vitro*. Immature DC differentiated into mature DC after loading with neoantigens. Cell morphology was observed with an inverted microscope and photographs were taken (**Figure 1**). In **Figure 1**, group A shows round monocytes with strong wall attachment after the removal of suspension cells on day D1. Group B shows adherent cells beginning to grow in clusters and extend pseudopodia on day D3. Group C shows a variety of cells with different morphology and pseudopodia on day D5. These can be characterized as immature dendritic cells, as the number of cells has significantly increased and some of the cells are suspended. Group D shows dendritic cells suspended after culture stimulation by CpG adjuvant and neoantigen 9aa peptides. They display short, transparent pseudopodia and are identified as mature dendritic cells. Group E shows the control group, no neoantigen 9aa peptides was added. The immature dendritic cells gradually die and break into cell fragments. After 7 days of PBMC culture, the morphology of DC in the group with neoantigens was significantly different from that without neoantigens (**Figure 2**).

### 3.3 The cellular immune effect induced by neoantigen peptides

Neoantigen peptides, as endogenous antigenic peptides, are phagocytized by antigen-presenting cells, such as DCs, before binding to corresponding HLA molecules presented on the cell surface. These complexes are recognized and bound by T-cell surface receptors, activating T-cells and promoting proliferation. Therefore, neoantigen peptides elicit a cellular immune response by promoting the maturation of DCs and activating T-cells upon binding to T-cell receptors (TCRs). In this study, the neoantigen peptide and CPG adjuvant synergistically acted on DCs to stimulate their maturation, allowing for the successful preparation of a DC vaccine loaded with neoantigen peptides and generating an effective T-cell immune response.

#### 3.3.1 Identification of mDCs via flow cytometry

**Figure 3:**
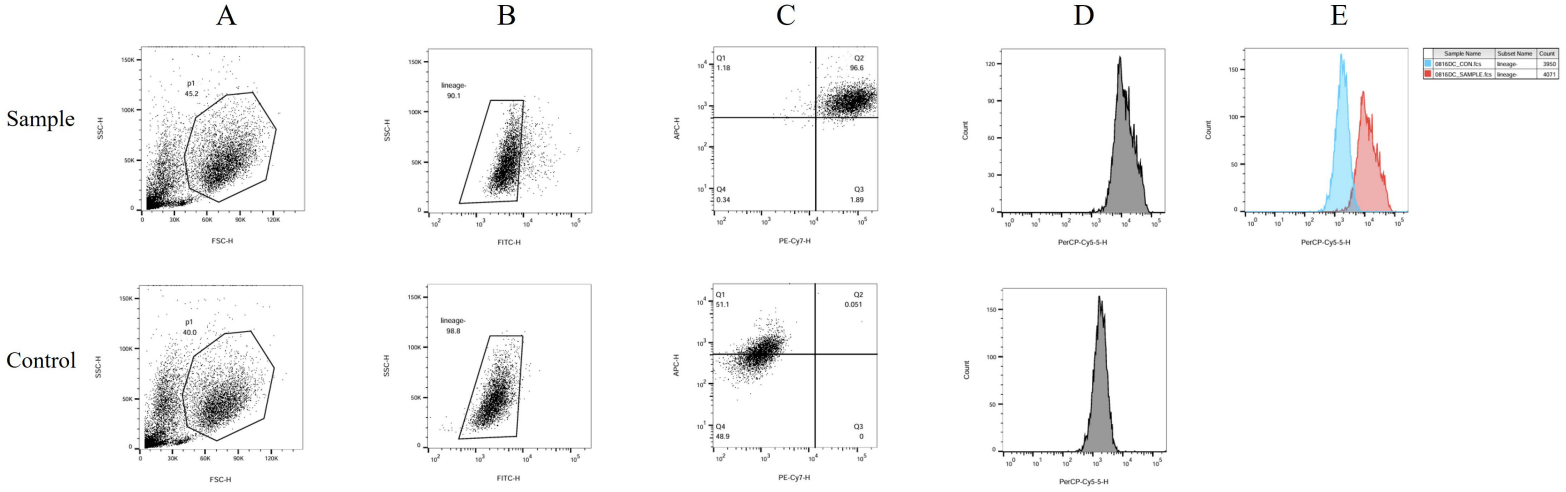
DC flow cytometry identification. The human peripheral blood by Ficoll density gradient centrifugation for peripheral blood mononuclear cell (PBMC), in vitro by GM-CSF and IL4 differentiation, add polypeptide and CPG adjuvant induced into mature dendritic cell (mDC). The sample group consisted of a polypeptide-induced cell population. The control group consisted of cells not exposed to polypeptides. The differences in CD11c, CD83, and CD83 positive expression between the two groups were compared. In this figure, **(A)** shows two distinct groups of cells. **(B)** was obtained by gating the P1 cell group in **(A)**. **(C**, **D)** were generated by gating part of the missing FITC lineage cell populations highlighted in **(B)** The cell population within Region Q2 in **(C)** is mDC expressing double positive for CD11c and CD83. Region Q2 constituted 96.6% of the Sample group and 0.051% of the Control group. The mDC content in the Sample group was significantly higher than in the Control group. **(D)** illustrates the single-parameter flow cytometry diagram of CD80 expression, while the comparison of CD80 fluorescence intensity in **(E)** demonstrates that the overall expression in the Sample group is significantly higher than in the Control group.

The human peripheral blood by Ficoll density gradient centrifugation for peripheral blood mononuclear cell (PBMC), *in vitro* by GM-CSF and IL4 differentiation, add polypeptide and CPG adjuvant induced into mature dendritic cell (mDC). The sample group consisted of a polypeptide-induced cell population. The control group consisted of cells not exposed to polypeptides. The differences in CD11c, CD83, and CD83 positive expression between the two groups were compared (**Figure 3**). In the **Figure 3**, **Figure 3A** shows two distinct groups of cells. **Figure 3B** was obtained by gating the P1 cell group in **Figure 3A**. **Figures 3C** and **D** were generated by gating part of the missing FITC lineage cell populations highlighted in **Figure 3B**. The cell population within Region Q2 in **Figure 3C** is mDC expressing double positive for CD11c and CD83. Region Q2 constituted 96.6% of the Sample group and 0.051% of the Control group. The mDC content in the Sample group was significantly higher than in the Control group. **Figure 3D** illustrates the single-parameter flow cytometry diagram of CD80 expression, while the comparison of CD80 fluorescence intensity in **Figure 3E** demonstrates that the overall expression in the Sample group is significantly higher than in the Control group.

#### 3.3.2 Investigating the percentages of T, B and NK cells in the final activated immune cells

**Figure 4:**
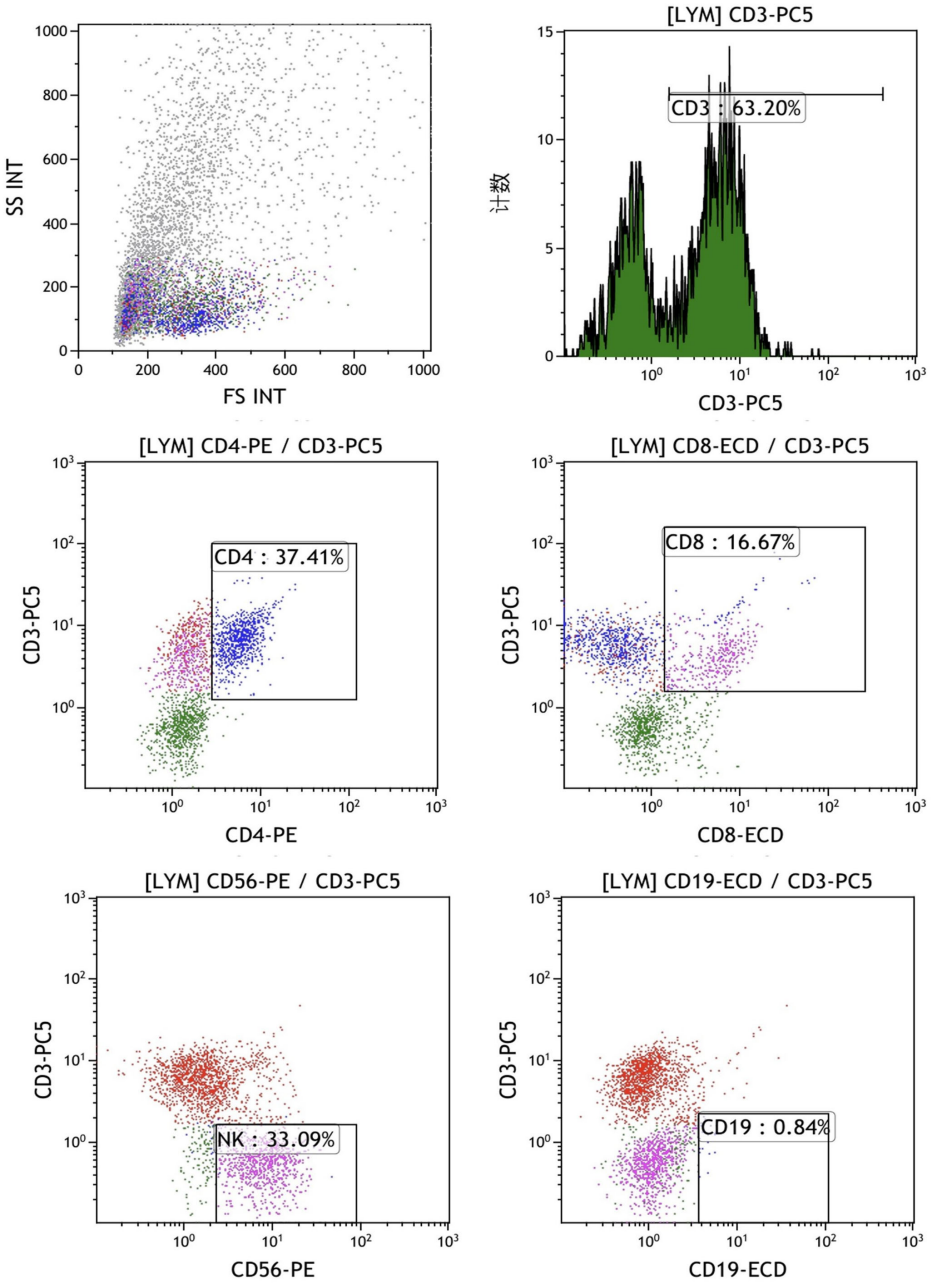
The percentages of T, B and NK cells in the mixed cells were identified by flow cytometry in experimental group shows the experimental group after adding peptides. The proportions of CD3+CD4+ double positive (CD4+ T cells), CD3+CD8+ double positive (CD8+ T cells), CD3-CD56 + (NK cells) and CD3-CD19 + (B cells) were 37.41%, 16.67%, 33.09% and 0.84%, respectively.

**Figure 5:**
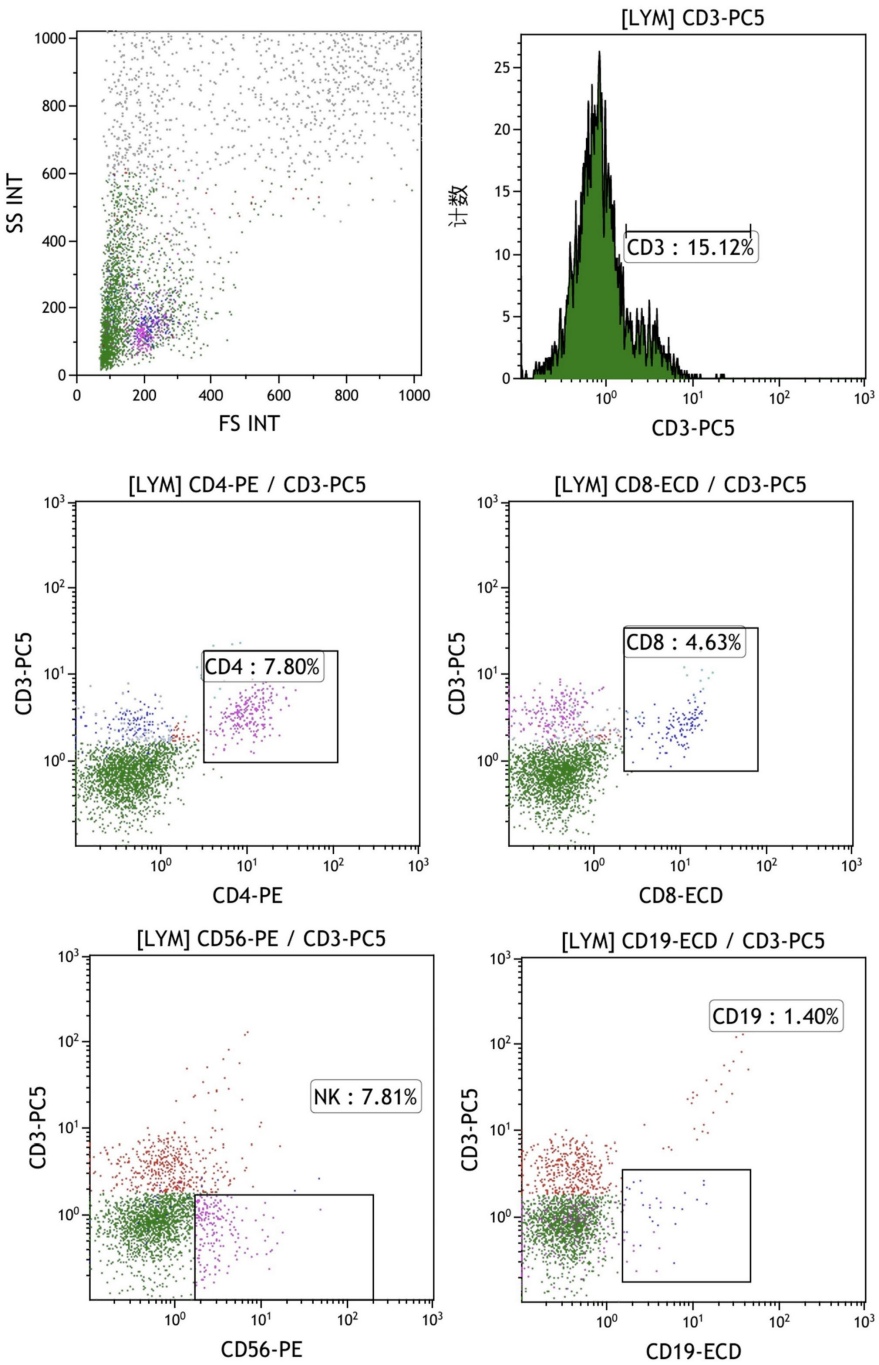
The percentages of T, B, and NK cells in the mixed cell population were analyzed by flow cytometry in the control group, which did not include the addition of peptides. The proportions of CD3+CD4+ double positive (CD4+ T cells), CD3+CD8+ double positive (CD8+ T cells), CD3-CD56 + (NK cells) and CD3-CD19 + (B cells) were 7.8%, 4.63%, 7.81% and 1.40%, respectively.

The final mixed culture cells after 14 days of culture were collected for flow cytometry analysis to show T, B, and NK cell percentages. **Figure 4** shows the experimental group after adding peptides. The proportions of CD3+CD4+ double positive (CD4+ T cells), CD3+CD8+ double positive (CD8+ T cells), CD3-CD56 + (NK cells) and CD3-CD19 + (B cells) were 37.41%, 16.67%, 33.09% and 0.84%, respectively. **Figure 4** shows the control group without adding peptides. The proportions of CD3+CD4+ double positive (CD4+ T cells), CD3+CD8+ double positive (CD8+ T cells), CD3-CD56 + (NK cells) and CD3-CD19 + (B cells) were 37.41%, 16.67%, 33.09% and 0.84%, respectively. **Figure 5** shows the control group without adding peptides. The proportions of CD3+CD4+ double positive (CD4+ T cells), CD3+CD8+ double positive (CD8+ T cells), CD3-CD56 + (NK cells) and CD3-CD19 + (B cells) were 7.8%, 4.63%, 7.81% and 1.40%, respectively. The proportion of CD4+T cells, CD8+T cells and NK cells in experimental group was significantly higher than that in control group. This suggests that neoantigen peptides induce proliferation not only of CD8+ T cells, but also of CD4+ T cells and NK cells simultaneously.

### 3.4 Neoantigen peptide ELISpot results

ELISpot assays were conducted after loading neoantigen peptides ZNF253 and ALPK1 at five different concentrations (10μg, 50μg, 100μg, 150μg, 200μg), respectively. The results (**Table 2**) indicated that the ELISPOT assay with a 100μg neoantigen loading dose produced the highest spot count, identifying 100μg as the optimal loading dose. Each spot represented an individual activated CD8+ T cell, and the spots were then quantified. Therefore, the immunogenicity of neoantigens can be quantified by spot counting to assess and compare the immunogenicity of different neoantigens. **Table 4** features the ELISpot results of neoantigen 9aa peptides from 6 colorectal cancer patients, showing the neoantigen 9aa peptide name and spot counts for each patient. Each spot corresponds to an activated CD8+ T cell, allowing for quantification of the immunogenicity of different neoantigens through spot counting.

**Table 4:** Chart and count of each patient's neoantigen ELISPOT.

Patient ID	Load dose of polypeptide/ Chart of ELISPOT /Spot count	Load dose of polypeptide/ Chart of ELISPOT /Spot count
Control	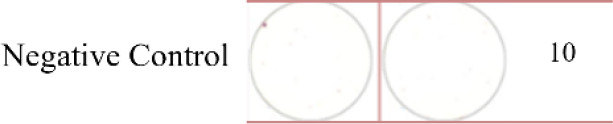	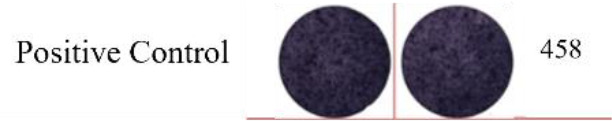
1	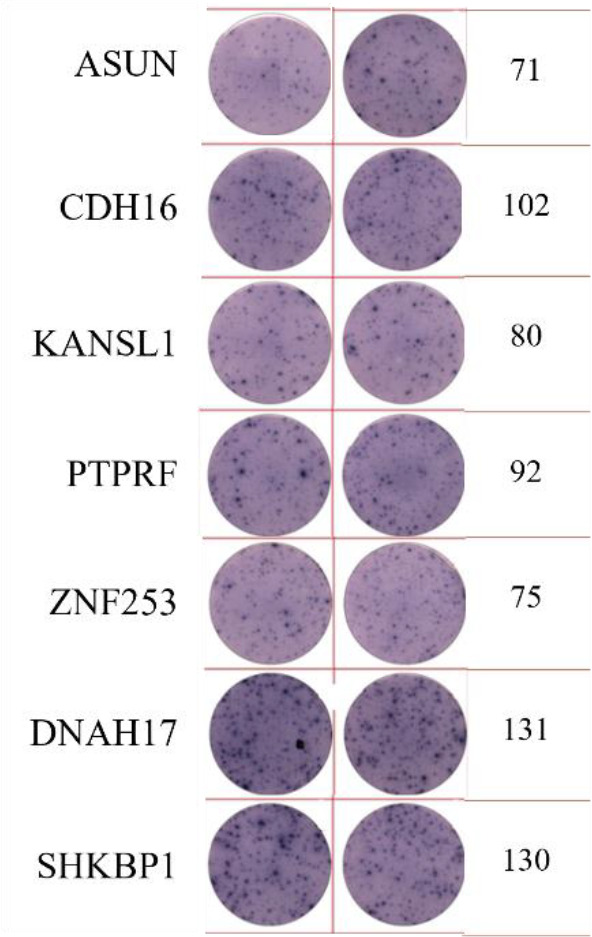	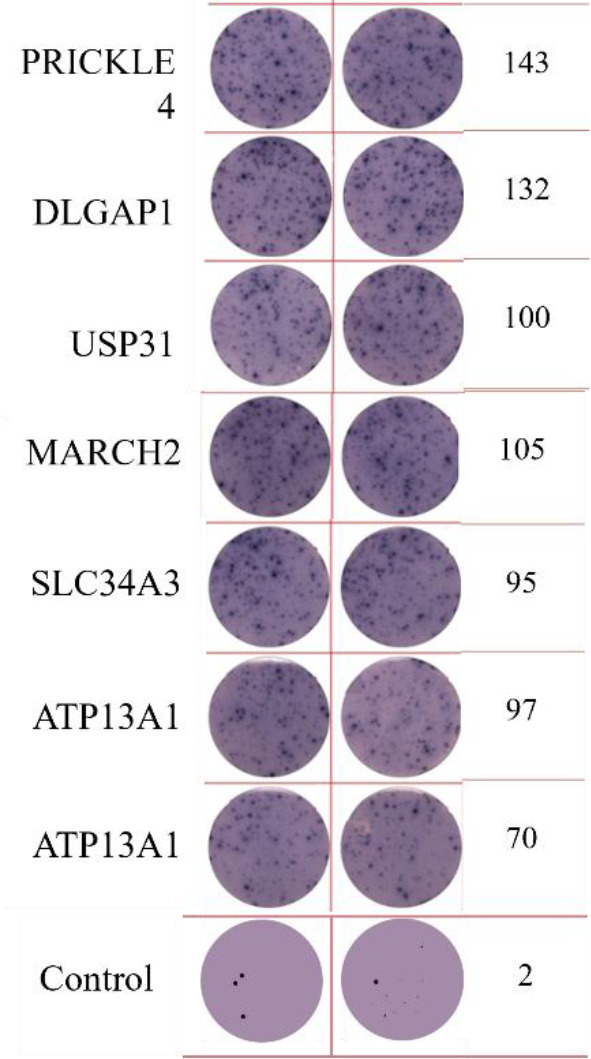
2	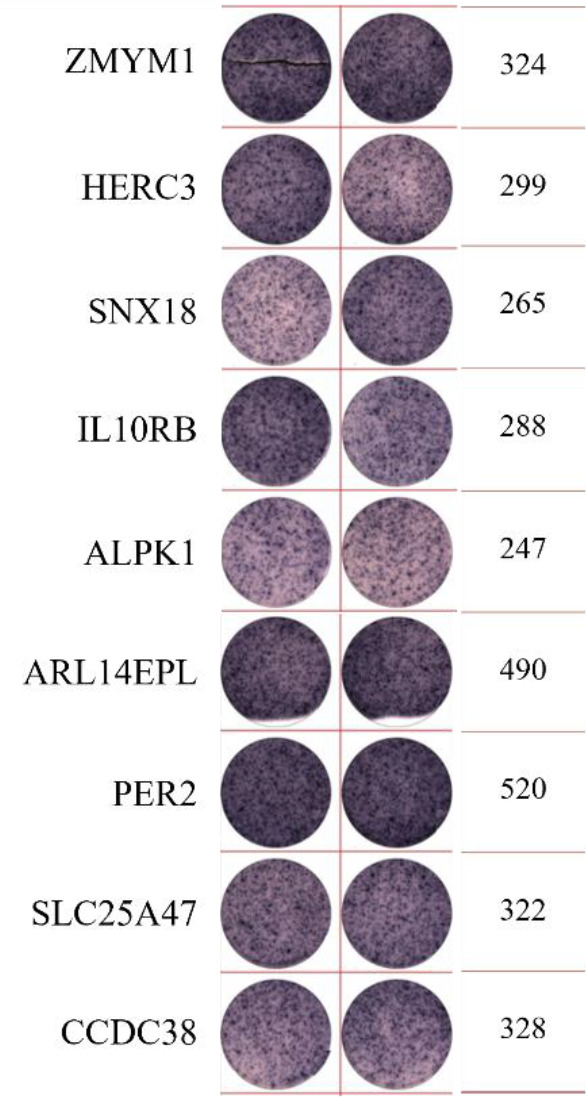	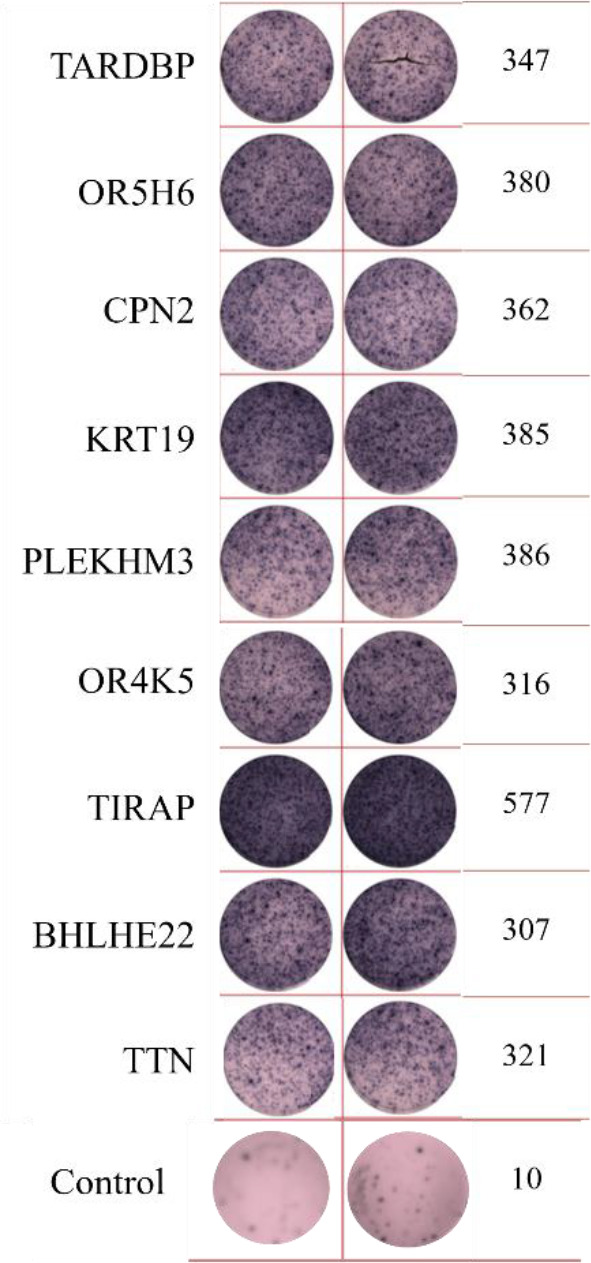
3	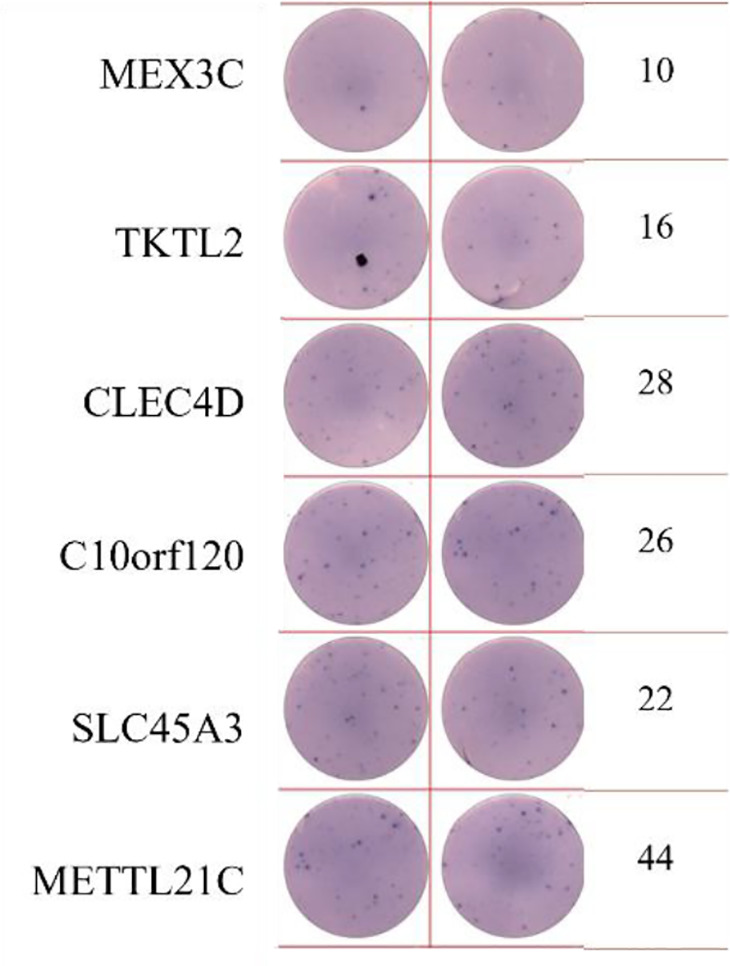	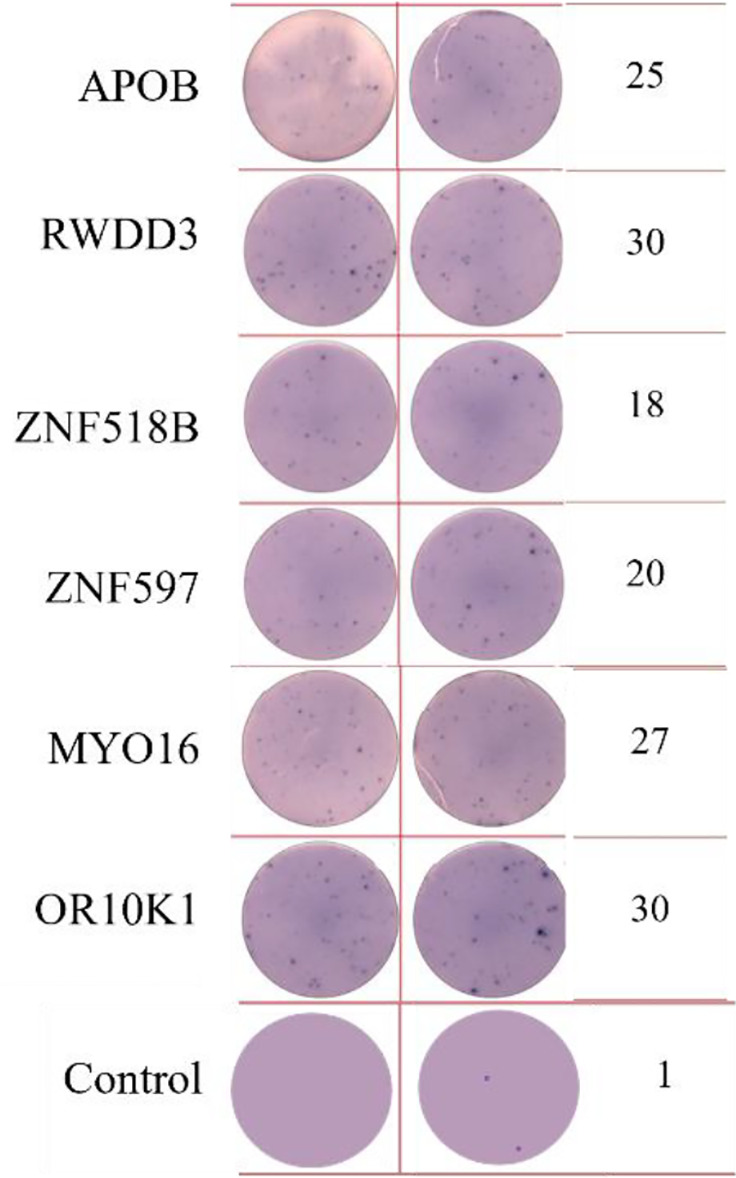
4	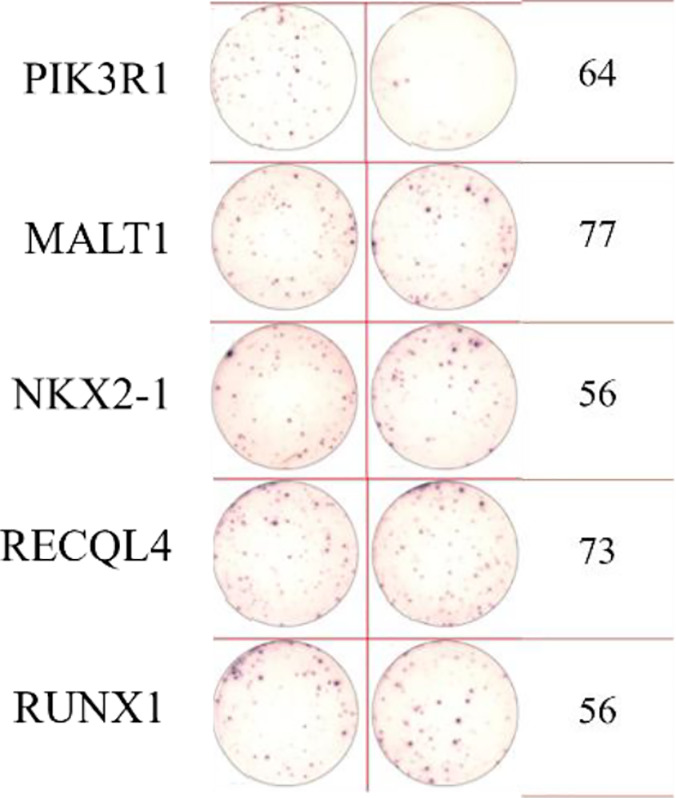	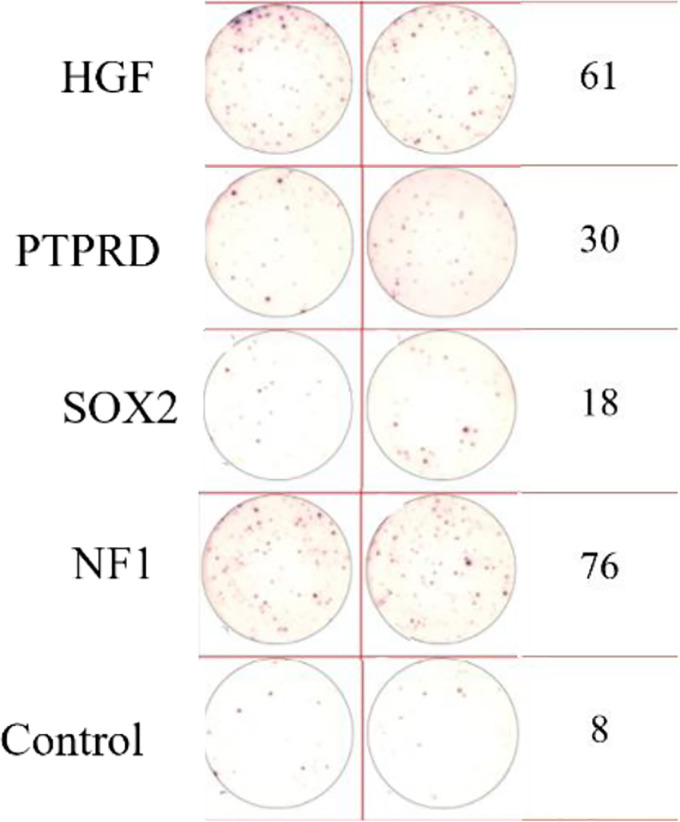
5	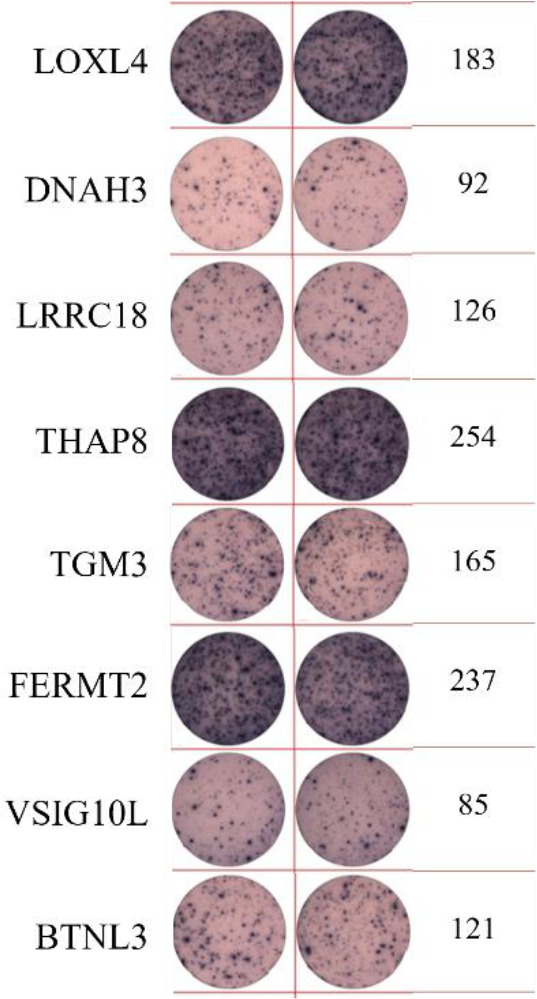	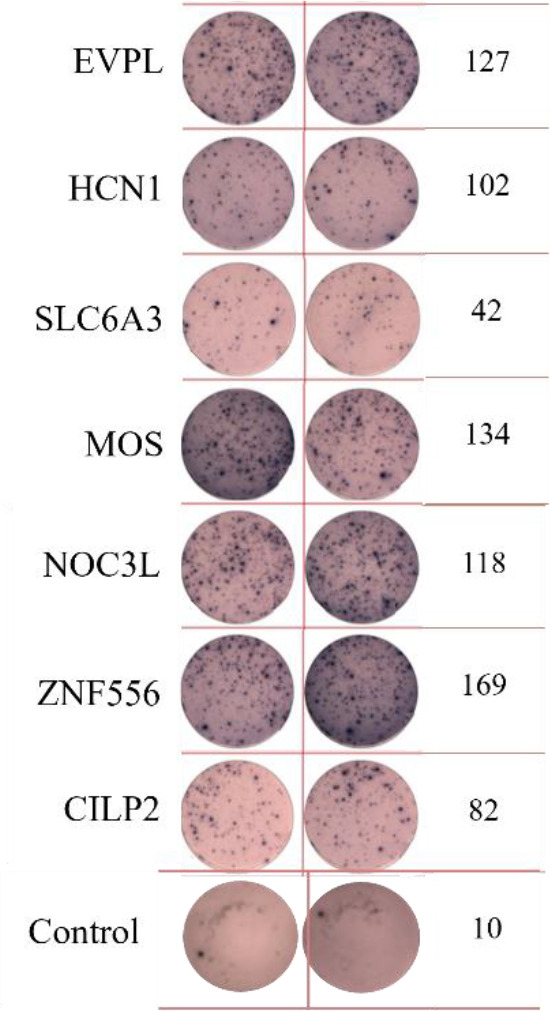
6	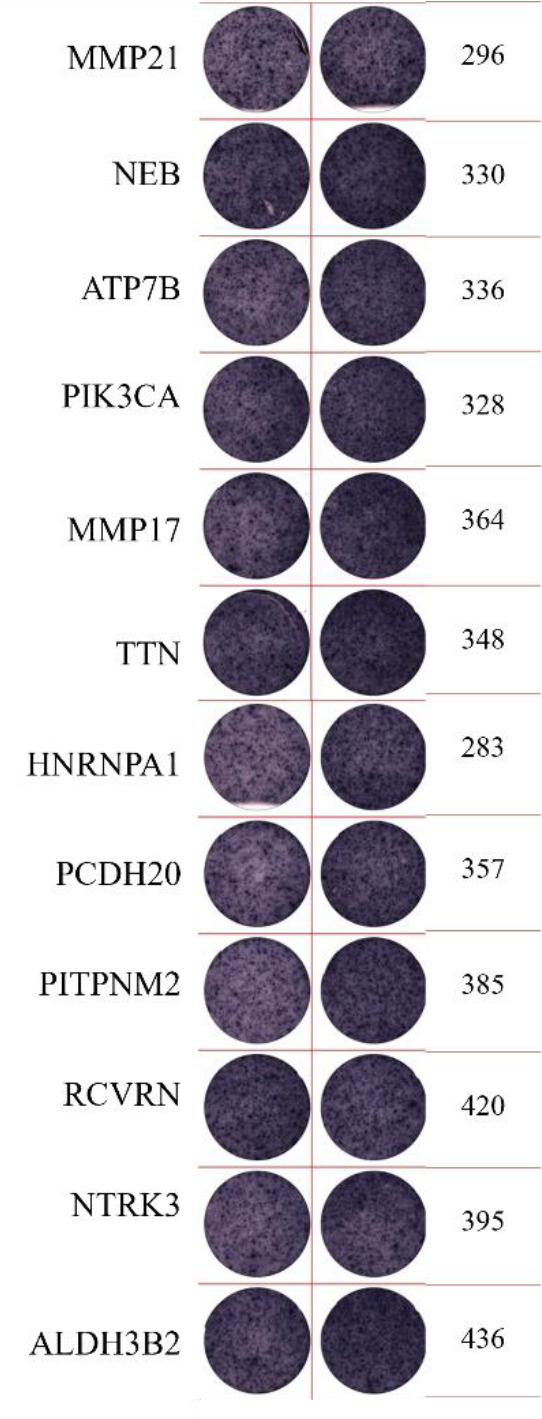	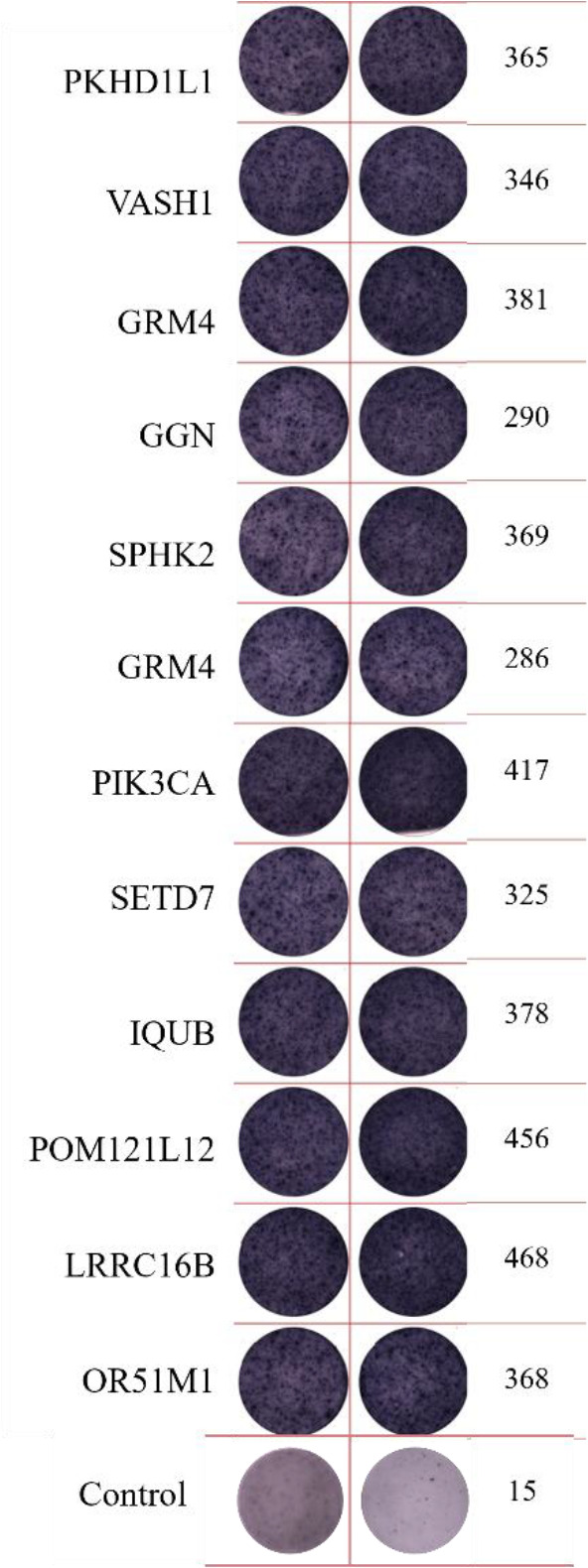

Associating the cellular immune effect of neoantigens (combined T value and cell subsets) with change in MHC molecular affinity and HLA-genotype.

(1) Each ELISpot represents an activated CD8+ T-cell, and the number of activated CD8+ T-cells indicates the immunogenicity of a neoantigen. Each ELISpot spot count for a new antigen was recorded as a T-value, with changes in neoantigen HLA molecular affinity calculated as the ratio of wild peptide HLA molecular affinity to that of the neoantigen. Similarly, changes in MHC molecular affinity are quantified as the ratio of wild peptide MHC molecular affinity to that of the neoantigens.

Immunogenicity quantification enabled determination of the relationship between a neoantigen’s immune effects and its HLA molecular affinity changes, as depicted in **Supplementary Material**. Numbers 1-6 on the chart represent individual patient identifiers. Each patient exhibits a distinct cellular immune response, implying unique baseline T-values. However, the changes in HLA molecular affinity for the peak values of each curve consistently fall within a similar range. The figure illustrates that neoantigens with the highest T-values exhibit HLA molecular affinity variations ranging from 1 to 4.

**Figure 6:**
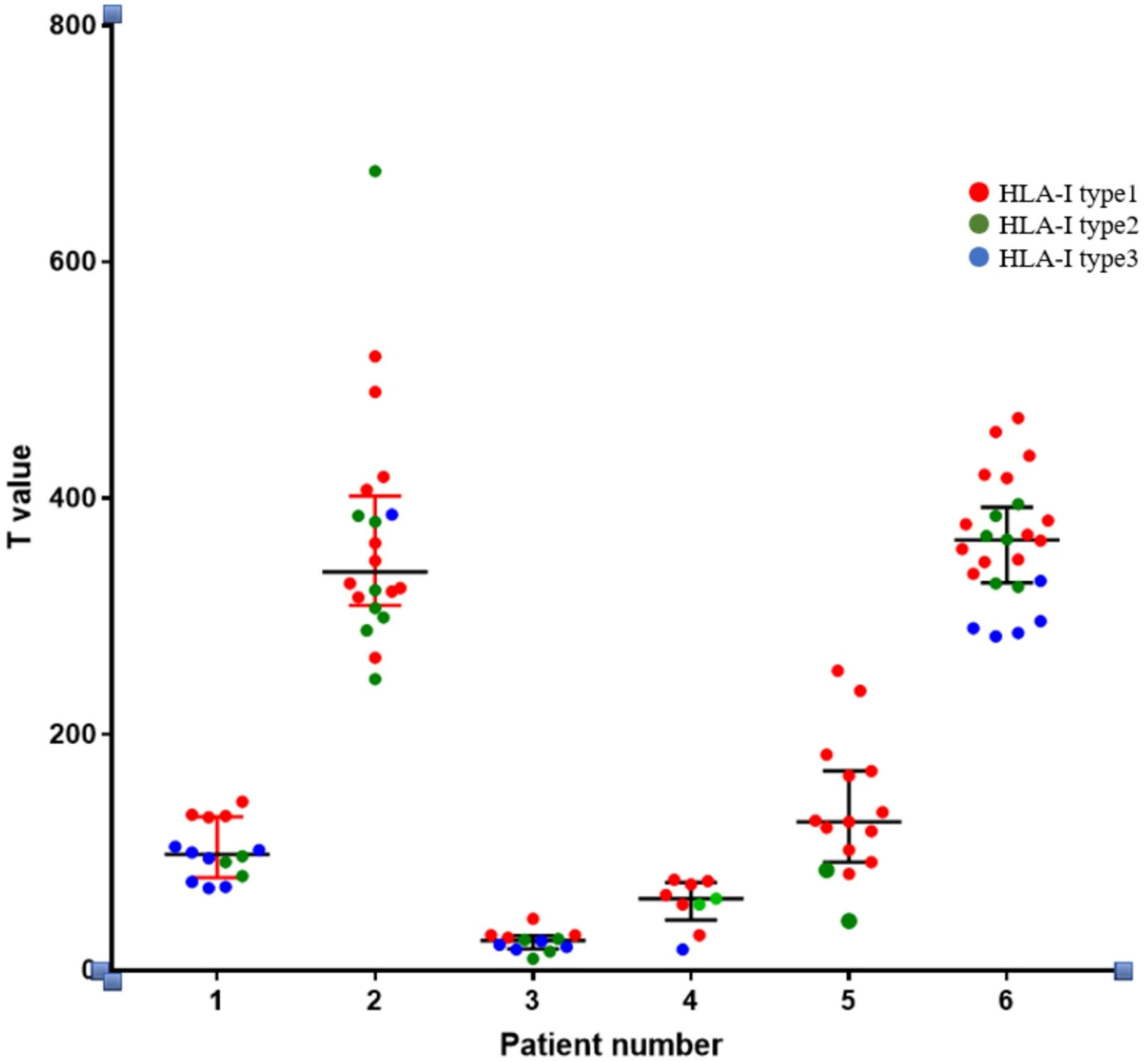
Relationship between the immunogenicity of neoantigenic polypeptides and HLA-allele genotypes. The neoantigens in each patient were typed. In the figure, it can be seen that in the HLA-I alleles of each patient, the largest number of binding neoantigen peptides was HLA-I typel, followed by HLA-I type2, and the smallest number of binding neoantigen peptides was HLA-I type3. We found that most of the most immunogenic neoantigens were HLA-I typel.

(2) Through whole-exome sequencing and HLA-I allele prediction, we identified that HLA molecules consistently bind to numerous neoantigens. Binding of HLA-I alleles to a larger repertoire of neoantigens correlates with increased immunogenicity of these neoantigens. Additionally, HLA Type-1 alleles exhibit the highest affinity for neoantigen peptides, followed by HLA Type-2 and Type-3 alleles. **Figure 6** illustrates the relationship between T-value and HLA ligand. Hence, neoantigens that bind to HLA Type-1 molecules exhibit the highest immunogenicity.

(3) Analysis:

1) Data conversion: The T-value of each patient was standardized and converted to the same dimensionality.

2) Two-factor grouping.

Two-factor grouping: HLA affinity changes were categorized into three groups: Group A (≤1), Group B (1-4), and Group C (>4).

HLA - I allele types were categorized into three groups for analysis. HLA Type 1 molecules bound to the highest number of neoantigen peptides, followed by HLA Types 2 and 3, respectively.

3) Statistical analysis results: Two-way ANOVA was conducted and presented in **Table 5**, alongside an interaction model analysis based on variations in HLA molecular affinity and HLA allelic genotype.

**Table 5:** Two-way ANOVA analysis of neoantigen standardized T levels for HLA affinity change interval and HLA-I allele type.

Descriptive statistics				
Dependent variable: Normalized T value				
Changes of HLA affinity (3 groups)	HLA-Iallele	Average	SD	N
≤1(Group A)	HLA-I type1	-.0563	.56550	12
	HLA-I type2	-.3642	.56286	7
	HLA-I type3	-.9846	.72423	8
	Sum	-.4111	.71380	27
1-4(Group B)	HLA-I type1	.7202	.97661	25
	HLA-I type2	.1324	1.04413	11
	HLA-I type3	-.5927	.72262	6
	Sum	.3787	1.05517	42
>4(Group C)	HLA-I type1	.2386	.72759	12
	HLA-I type2	-.7785	.77795	7
	HLA-I type3	-.5549	.54876	4
	Sum	-.2089	.83968	23
Sum	HLA-I type1	.4121	.88505	49
	HLA-I type2	-.2617	.91484	25
	HLA-I type3	-.7585	.68280	18
	Sum	.0000	.97214	92

Results of intersubjective effect test: The change of the HLA molecular affinity degree * HLA - I allele type: F = 0.762, *p* = 0.553, which suggests that there is no interaction between the change in HLA molecular affinity degree and the genotype of the HLA alleles.

The results were analyzed via two-way ANOVA: In comparison of HLA molecular affinity variation ranges (**Table 6**), the standardized T-value corresponding to a HLA molecular affinity variation range of 1-4 (Group B) is significantly higher than those for ≤1 (Group A, *p* < 0.0001) and >4 (Group C, *p* < 0.05), enough to be considered statistically significant. In the comparison of HLA-allele genotypes (**Table 7**), the standardized T-value of HLA-Type 1 was significantly higher than those of HLA-Type 2 (*p* < 0.05) and HLA-Type 3 (*p* < 0.0001). The HLA allele of the neoantigen with the highest T-value was found to be HLA-Type 1.

**Table 6:** Multiple comparisons of HLA affinity grouping.

Dependent variable: Normalized T value					
	(I) Changes of HLA affinity	(J) Changes of HLA affinity	The difference in the mean (I-J)	SD	*P* value
LSD	≤1(Group A)	1-4(Group B)	-.7899*	.20235	.000
		>4(Group C)	-.2022	.23277	.388
	1-4(Group B)	≤1(Group A)	.7899*	.20235	.000
		>4(Group C)	.5877*	.21279	.007
	>4(Group C)	≤1(Group A)	.2022	.23277	.388
		1-4(Group B)	-.5877*	.21279	.007

Based on measured mean value.

Mean Squared Error=0.673.

*The significance level of the mean difference was p<0.05.

**Table 7:** Multiple comparisons of HLA-Iallele type grouping.

Dependent variable: Normalized T value					
	(I) HLA-Iallele	(J) HLA-Iallele	The difference in the mean (I-J)	SD	*P* value
LSD	HLA-I type1	HLA-I type2	.6738*	.20162	.001
		HLA-I type3	1.1706*	.22609	.000
	HLA-I type2	HLA-I type1	-.6738*	.20162	.001
		HLA-I type3	.4968	.25358	.053
	HLA-I type3	HLA-I type1	-1.1706*	.22609	.000
		HLA-I type2	-.4968	.25358	.053

Based on measured mean value.

Mean Squared Error =0.673.

*The significance level of the mean difference was p<0.05.

## 4 Discussion

In recent years, advancements in bioinformatics have catalyzed significant progress in personalized tumor vaccines. Examination of tumor mutations enables testing of immune responses to mutant proteins, or neoantigens, through computer simulations. This approach facilitates screening for immunogenic neoantigens and the development of polypeptide or mRNA tumor vaccines for clinical trials. Whole exome sequencing was performed on tumor tissue and matched healthy tissue (typically peripheral blood), followed by comparison to identify somatic gene mutations. This method typically identifies the majority of single nucleotide variants with sufficient coverage. In 2013, Rosenberg et al. pioneered whole exome sequencing to identify mutated proteins in patients. They utilized an MHC molecule-epitope affinity algorithm to predict and synthesize candidate epitopes for immune response validation. This approach enables rapid identification of mutated antigens expressed on tumor cells, facilitating their recognition by tumor-infiltrating lymphocytes (TIL). Subsequently, this method was clinically applied in 2014, again by Rosenberg’s team. High-depth exon sequencing technology and immune response verification enabled identification of a high-frequency mutant gene in a patient with metastatic cholangiocarcinoma, along with its corresponding TIL clone. Amplification of the TIL clone and retrotransfusion therapy effectively controlled the patient’s disease (10). Also in 2013, another team utilized exon sequencing technology alongside transcriptome sequencing and high-throughput proteomic analysis to predict MHC molecule-antigen epitope affinity, facilitating T cell recognition and efficient activation peptide vaccine production. This personalized tumor vaccine demonstrated efficacy comparable to both preventive and therapeutic vaccines (11). With the discovery of additional HLA alleles, software tools like NetChop, NetMHCpan, NetMHC, and SYFPEITHI have been developed to predict MHC molecular ligands of neoantigen peptides and their affinities. However, due to variations in neoantigens between patients and their limited immunogenicity, this discovery process is complex and time-consuming (12, 13). Certain immunoeffective neoantigens induce immune tolerance, impotence, and even immunosuppression. These factors hinder the clinical application of personalized vaccines, underscoring the need to rapidly identify effective neoantigen peptides and enhance the immune response of those with weak immunogenicity (5, 14).

Currently, many algorithms are capable of predicting the affinity between peptides and MHC molecules. However, the MHC affinity of peptides does not correlate with their immunogenicity; it is believed that immunogenicity is more closely associated with the stability of the peptide-MHC complex (15, 16). Kristensen et al. demonstrated that predicting the stability of binding between peptides and MHC molecules can increase the hit rate of candidate neoantigens (i.e., successful predictions of immunoeffective neoantigens) (17). Assarsson et al. conducted a systematic analysis of putative and actual vaccinia virus epitopes, determining that epitope dominance had little relationship with HLA peptide affinity, stability, TCR affinity, or the number of processed epitopes (18). Another study suggested that the NETMHC score was not a valid predictor of immunogenicity or tumor rejection. The authors developed the DAI algorithm, which subtracts the NETMHC scores of unmutated copies of predicted mutated epitopes from those of the corresponding mutated epitopes, accounting for the NETMHC scores of those copies. Although the DAI algorithm identified far more tumor-protective epitopes than those derived from the highest NETMHC or MHC-binding scores, most epitopes identified by DAI still fail to elicit tumor protection (9).

Peptides can serve directly as T-cell epitopes due to low toxicity, precise targeting, cost-effectiveness, and ease of chemical synthesis, rendering them appealing for vaccine development. Peptides bound to MHC class I molecules are typically 8-11 amino acids long, reflecting their shorter gene sequence (19). Conversely, MHC class II molecules feature open grooves at both ends, accommodating longer peptides ranging from approximately 10 to 30 amino acids. Consequently, they possess more complex binding motifs and bind a broader range of exogenous antigenic peptides compared to MHC class I molecules. Moreover, neoantigens are endogenous and structurally straightforward, simplifying the study of peptides bound to MHC class I molecules compared to those acting as MHC class II antigens. The most prevalent peptide segment length is 9 amino acids, making it the most specific MHC binding ligand (20). *In vitro* synthesis of neoantigenic peptides can elicit antigen-specific T-cell responses (19). In this study, the 9 amino acid peptide mutant sequence was identified through whole exon sequencing, synthesized *in vitro*, and evaluated for antigen-specific T-cell responses. Dendritic cells, as antigen-presenting cells (APCs) with potent antigen-presenting capabilities, can present long peptides to immature T-cells for activation. Other APCs may present short peptides, but these can lead to anergy in stimulated T-cells due to the absence of a co-stimulatory molecule (21). Provenge (Sipuleucel-T), utilizing *in vitro* dendritic cells, received FDA approval in 2010 as the first therapeutic prostate cancer vaccine. Short peptides like neoantigen 9 amino acid peptides require effective adjuvants, such as TLR9 or TLR3 agonists, to induce an effective CD8+ T-cell response, mediated through DC activation and immune system stimulation (22). A study compared the effects of CpG ODN adjuvant and Freund’s adjuvant in combination with a tumor vaccine. CpG ODN demonstrated superior adjuvant effects over Freund’s adjuvant, with lower associated toxicity, highlighting its utility as a tumor vaccine adjuvant (23).

Granulocyte-macrophage colony-stimulating factor (GM-CSF) induces PBMC differentiation into macrophages and dendritic cells, while interleukin 4 (IL4) inhibits macrophage formation (24). Thus, dendritic cells can be prepared *in vitro* by isolating PBMCs from peripheral blood and culturing them in a medium supplemented with GM-CSF and IL4 cytokines. Dendritic cells prepared in this manner are referred to as immature dendritic cells (imDCs) (25, 26). In this study, DCs were loaded with neoantigen peptides to prepare DC vaccines by promoting DC maturation. The expression of marker molecules such as CD11c, CD80, CD86, and CD83, indicative of dendritic cell maturation, was assessed using flow cytometry. Detection results indicated that mature dendritic cell (mDC) levels in cell suspensions of DC vaccines exceeded 97%.

Peptides are easy to produce, allowing multiple sequences to be combined into a single formulation, thereby enabling direct monitoring of the vaccine’s immune effects through its individual components. Less than 10% of tumor cell gene mutations give rise to tumor antigens, and fewer than 10% of these neoantigens can successfully induce effective, specific T-cell immunity (the primary anticancer immunity) (8). Whole exon sequencing identifies a large number of potential neoantigen peptides, necessitating screening for those with high immunogenicity. Higher MHC molecule affinity increases the likelihood of becoming neoantigens; however, MHC affinity does not correlate with neoantigen immunogenicity. In a 2017 Nature study on neoantigen vaccines for melanomas (5), only neoantigen peptides with a substantial change in MHC affinity were included, showing a significant therapeutic effect. However, this does not constitute evidence of a broad correlation between immunogenicity and MHC affinity, and no relevant studies have confirmed such a relationship. This study confirmed that neoantigen immunogenicity did not correlate positively with changes in MHC class I molecular affinity. Only neoantigens with a significant change in MHC affinity were selected as polypeptide vaccines, leading to a high rate of misselection and omission.

This study utilized whole exon sequencing results from tumor tissues and predictions of MHC class I molecular affinity to screen for neoantigenic 9aa peptides. These peptides were combined with CPG adjuvant *in vitro* to simulate endogenous antigen presentation by APCs, specifically dendritic cells, to CD8+ T-cells, thereby activating their immunological response. Immunogenicity of each antigen was quantified using ELISpot, an enzyme-linked spot experiment. We observed that the immunogenicity of neoantigens did not show a positive correlation with the variation in affinity of MHC Class I molecules. The MHC affinity variation among neoantigens with the highest immunogenicity mostly ranged between 1 and 4, and the HLA-I genotype associated with the highest T-values predominantly exhibited binding to the greatest number of neoantigen peptides.

Neoantigenic 9aa peptides, as endogenous antigens, can specifically activate and proliferate CD8+ T-cells. However, several studies have demonstrated their capability to activate CD4+ T-cells as well (27–30). In this study, a DC vaccine was co-cultured with PBMCs. Flow cytometry analysis of T, B, and NK cells indicated that the neoantigen peptide not only activated CD8+ T-cells but also contributed to the proliferation of CD4+ T-cells and NK cells. This suggests that neoantigen peptides can activate both specific (CD8+ T-cell) and nonspecific (CD4+ T-cell and NK cell) immunity.

This study has helped elucidate the relationship between neoantigen immunogenicity and changes in MHC molecular affinity and HLA alleles, providing a novel method for rapid screening of strongly-immunoeffective neoantigens.

## 5 Conclusions

1. The most immunogenic neoantigens typically exhibit an MHC molecular affinity variation between 1 and 4, indicating that stronger immunogenicity correlates with higher MHC molecular affinity variation.
2. Each patient’s HLA molecules were classified into Types 1, 2, and 3, with Type 1 showing the highest binding capacity for neoantigens. Our findings indicate that the most immunogenic neoantigens were associated with HLA Type 1.
3. Neoantigen peptides were shown to activate the proliferation of both CD8+ T-cells and induce proliferation of CD4+ T-cells and NK cells.
4. Variation in MHC molecular affinity and HLA neoantigen genotype are anticipated to serve as valuable variables for screening highly immunogenic neoantigens, facilitating more efficient preparation of effective polypeptide tumor vaccines.

## Data Availability

The datasets presented in this article are not readily available due to the need for information confidentiality. Requests to access the datasets should be directed to the corresponding author.
