# Corrigendum: Anti-cancer immune effect of human colorectal cancer neoantigen peptide based on MHC class I molecular affinity screening

**DOI:** 10.3389/fimmu.2024.1514836

**Published:** 2024-12-03

**Authors:** Siyu Zhang, Changxin Huang, Yongqiang Li, Zhaoyang Li, Ying Zhu, Lili Yang, Haokun Hu, Quan Sun, Mengmeng Liu, Songqiang Cao

**Affiliations:** ^1^ Department of Oncology, Affiliated Hospital of Hangzhou Normal University, Hangzhou, China; ^2^ Department of Clinical Hematology and Transfusion, The First Affiliated Hospital of Zhejiang Chinese Medical University, Hangzhou, China; ^3^ Department of Psychiatry and Psychology, 155 Hospital of Kaifeng City, Kaifeng, China; ^4^ Department of Urology, Huaihe Hospital of Henan University, Kaifeng, China

**Keywords:** tumor immunotherapy, tumor vaccine, neoantigen, MHC molecular affinity, colorectal cancer

In the published article, there was two error in affiliation(s) 2.

1. Instead of “^3^Department of Psychiatry and Psychology, 155 Hospital of Kaifeng City, Hangzhou, China”, it should be “^3^Department of Psychiatry and Psychology, 155 Hospital of Kaifeng City, Kaifeng, China”.

2. Instead of “^4^Department of Urology, Huaihe Hospital, Henan University, Kaifeng City, China”, it should be “^4^Department of Urology, Huaihe Hospital of Henan University, Kaifeng, China”.

In the published article, there was an error in the legend for **Figure 5** as published. “The percentages of T, B and NK cells in the mixed cells were identified by flow cytometry in control group shows the experimental group after adding peptides. The proportions of CD3+CD4+ double positive (CD4+ T cells), CD3+CD8+ double positive (CD8+ T cells), CD3-CD56 + (NK cells) and CD3-CD19 + (B cells) were 7.8%, 14.63%, 7.81% and 1.40%, respectively” The corrected legend appears below.

**Figure 5:**
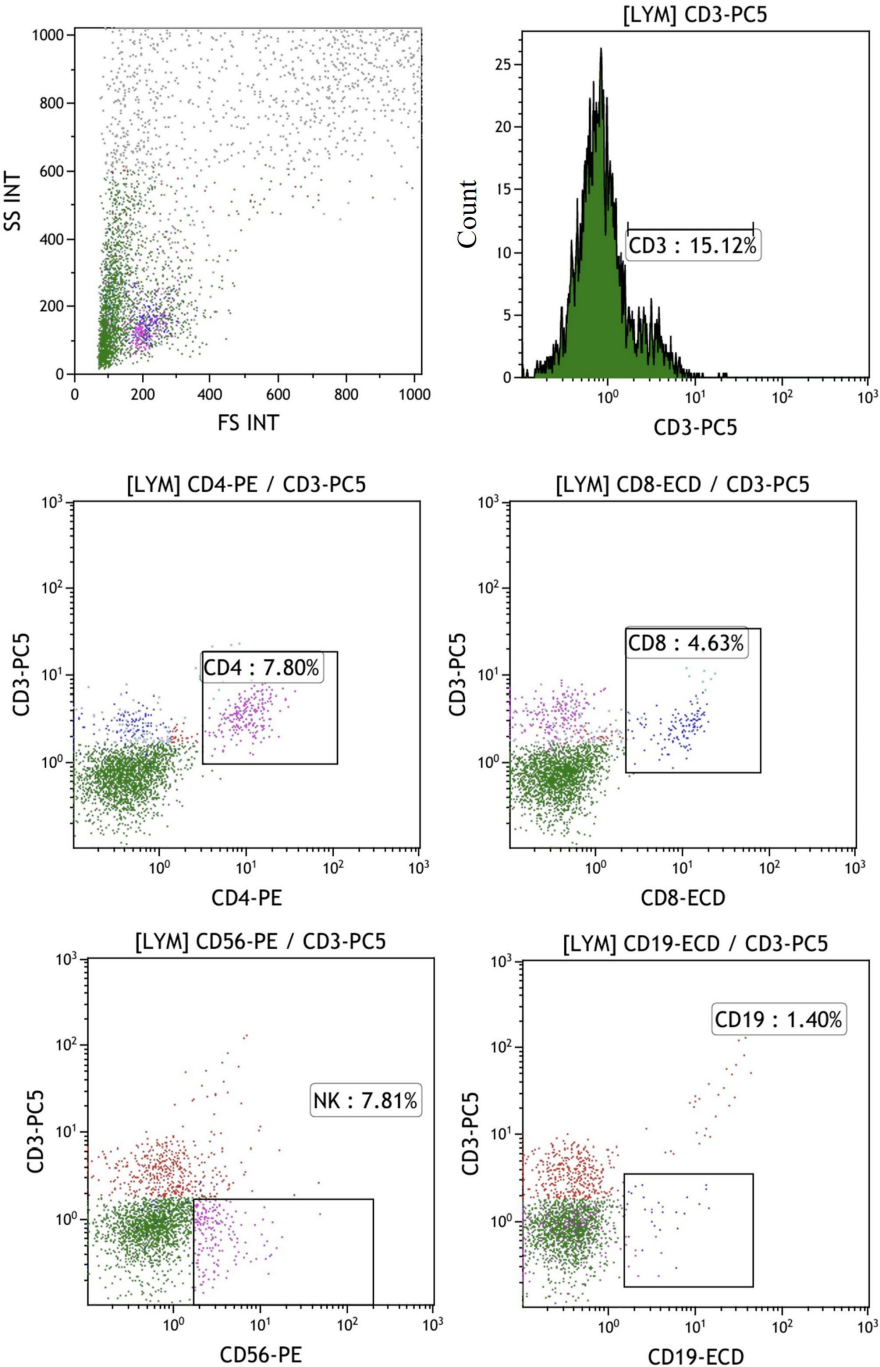
The percentages of T, B, and NK cells in the mixed cell population were analyzed by flow cytometry in the control group, which did not include the addition of peptides. The proportions of CD3+CD4+ double positive (CD4+ T cells), CD3+CD8+ double positive (CD8+ T cells), CD3-CD56 + (NK cells) and CD3-CD19 + (B cells) were 7.8%, 4.63%, 7.81% and 1.40%, respectively.

“The percentages of T, B, and NK cells in the mixed cell population were analyzed by flow cytometry in the control group, which did not include the addition of peptides. The proportions of CD3+CD4+ double positive (CD4+ T cells), CD3+CD8+ double positive (CD8+ T cells), CD3-CD56 + (NK cells) and CD3-CD19 + (B cells) were 7.8%, 4.63%, 7.81% and 1.40%, respectively.”

In the published article, there was an error in the **Funding** statement. “This work was supported by grants from major science and technology project of Zhejiang Province, China (Project number: 4125C4011724448), the Hangzhou Science and Technology Development Program Project (Project number: 202004A21), Hangzhou Medical and Health Technology Project (Project number: A20220868), Hangzhou Biomedicine and Health Industry Development Support Project (Project number: 2022WJC030), and Henan Province Medical Science and Technology Research Program Project (Project number: LHGI20230440).” The correct Funding statement appears below.

“This work was supported by grants from major science and technology project of Zhejiang Province, China (Project number: 2017C03053), the Hangzhou Science and Technology Development Program Project (Project number: 202004A21), Hangzhou Medical and Health Technology Project (Project number: A20220868), Hangzhou Biomedicine and Health Industry Development Support Project (Project number: 2022WJC030), and Henan Province Medical Science and Technology Research Program Project (Project number: LHGI20230440).”

In the published article, there were three errors.

1. A correction has been made to **Abstract**, *Results*, 1. This sentence previously stated:

“3. Neoantigen Peptides Promote CD4+, CD8+ T, and NK Cell Proliferation: After 14 days, flow cytometry showed higher percentages of CD4+ T (37.41% vs 7.8%), CD8+ T (16.67% vs 14.63%), and NK cells (33.09% vs 7.81%) in the experimental group, indicating that the neoantigen peptides induced proliferation of CD4+, CD8+ T cells, and NK cells.”

The corrected sentence appears below:

“3. Neoantigen Peptides Promote CD4+, CD8+ T, and NK Cell Proliferation: After 14 days, flow cytometry showed higher percentages of CD4+ T (37.41% vs 7.8%), CD8+ T (16.67% vs 4.63%), and NK cells (33.09% vs 7.81%) in the experimental group, indicating that the neoantigen peptides induced proliferation of CD4+, CD8+ T cells, and NK cells.”

2. A correction has been made to *3.3 The cellular immune effect induced by neoantigen peptides*, *3.3.2 Investigating the percentages of T, B and NK cells in the final activated immune cells*, 1. This sentence previously stated:

“The proportions of CD3+CD4+ double positive (CD4+ T cells), CD3+CD8+ double positive (CD8+ T cells), CD3-CD56 + (NK cells) and CD3-CD19 + (B cells) were 7.8%, 14.63%, 7.81% and 1.40%, respectively.”

The corrected sentence appears below:

“The proportions of CD3+CD4+ double positive (CD4+ T cells), CD3+CD8+ double positive (CD8+ T cells), CD3-CD56 + (NK cells) and CD3-CD19 + (B cells) were 7.8%, 4.63%, 7.81% and 1.40%, respectively.”

3. A correction has been made to **3 Results**, *3.4 Neoantigen peptide ELISpot results*, 4. This sentence previously stated:

“Immunogenicity quantification enabled determination of the relationship between a neoantigen’s immune effects and its HLA molecular affinity changes, as depicted in Chart 1.”

The corrected sentence appears below:

“Immunogenicity quantification enabled determination of the relationship between a neoantigen’s immune effects and its HLA molecular affinity changes, as depicted in Supplementary Material.”

The authors apologize for these errors and state that these do not change the scientific conclusions of the article in any way. The original article has been updated.

